# Loss of cerebral autoregulation drives abnormal perfusion patterns after stroke

**DOI:** 10.1073/pnas.2534566123

**Published:** 2026-09-29

**Authors:** Chryso Lambride, Mohamad El Amki, Susanne Wegener, Franca Schmid

**Affiliations:** ^a^ Department of Neurology, University Hospital and University of Zurich, Zurich 8057, Switzerland; ^b^ Neuroscience Center Zurich, University of Zurich and ETH Zurich, Zurich 8091, Switzerland; ^c^ ARTificial ORGan Center for Biomedical Engineering Research, University of Bern, Bern 3010, Switzerland

**Keywords:** cerebral autoregulation, computational modeling, cerebral blood flow, microcirculation, ischaemic stroke

## Abstract

Maintaining a stable cerebral blood flow (CBF) despite variations in blood pressure is crucial for normal brain function. This process, called cerebral autoregulation, is often impaired in pathologies such as ischemic stroke, underlining its clinical importance. However, how local vascular responses interact across complex cerebrovascular networks to stabilize perfusion remains poorly understood. Here, we present an in silico model of static autoregulation that can be applied to realistic microvascular networks, providing vessel-specific insights into vasoreactivity and perfusion dynamics. Our simulations revealed a hierarchical organization of autoregulatory responses, in which pial surface arteries act as key regulators that buffer pressure changes across large vascular territories. Arterial density emerged as a decisive determinant of network-specific autoregulatory capacity, highlighting the importance of vascular topology for maintaining stable perfusion. During poststroke reperfusion, alterations in vasoreactivity, rather than collateral extent alone, emerged as the main contributor to pathogenic hyperperfusion. Simulations of chronic autoregulatory dysfunction showed that progressive loss of vascular reactivity can produce substantial downstream disturbances in capillary perfusion and alter the autoregulation curve. Overall, our framework reveals how interactions between vascular topology and vessel-specific regulatory mechanisms shape CBF regulation across scales, from individual arteries to whole-network perfusion. These findings highlight myogenic tone as a potential therapeutic target to reduce reperfusion-related complications in stroke.

## Significance

Cerebral autoregulation is essential for maintaining stable brain perfusion, yet the vascular mechanisms that coordinate blood flow regulation across cerebrovascular networks remain incompletely understood. Using an in silico framework applied to realistic microvascular networks, we show that autoregulation emerges from coordinated responses throughout the arterial network, with surface arteries acting as key regulators of global blood flow, and arterial density determining the resilience to pressure perturbations. We further demonstrate that impaired regulation after stroke substantially alters flow redistribution during reperfusion and may contribute to detrimental hyperperfusion in some patients following vessel recanalization. These findings provide mechanistic insight into how local vascular dysfunction propagates through networks and identify myogenic tone preservation as a potential therapeutic target to mitigate reperfusion-related injury.

Cerebral autoregulation is a key physiological process that maintains cerebral blood flow (CBF) relatively constant across a range of blood pressures, primarily through adjustments in arterial diameter (1, 2). This regulation is essential for normal brain function and relies on a complex interplay of vascular control mechanisms. Although the underlying mechanisms are not yet fully elucidated, current evidence suggests vascular smooth muscle (VSM) tone as the primary driver of CBF regulation (3). Given their high density of VSM cells (VSMCs), arteries stand out as key regulators for vascular tone in response to pressure changes (4, 5).

Both VSMCs and endothelial cells contribute critically to this regulation (2, 6–9), largely due to their mechanosensitive properties, which allow them to respond to changes in transmural pressure and shear stress (7, 9). The myogenic response, driven intrinsically by VSMCs, involves vasoconstriction in response to elevated intravascular pressure and is considered independent of neural, metabolic, or hormonal influences (2, 3). On the other hand, the endothelial (flow-mediated) response is triggered by increased shear stress, leading to the release of vasoactive signals that promote VSM relaxation and vasodilation (2, 10). The net effect on vascular tone reflects the balance between these opposing mechanisms and is mediated via the degree of VSM myosin light chain phosphorylation (11, 12).

While many studies have focused on cerebral autoregulatory mechanisms (4, 5, 13–17), the contribution of individual arteries and the resulting flow changes at single-vessel resolution remain largely unexplored. Quantifying single-vessel responses in vivo is particularly challenging due to the concurrent activity of multiple physiological processes and the large spatial scale, ranging from the smallest to the largest vessels, that needs to be resolved. To address this, we developed a static autoregulation model incorporating pressure- and flow-dependent responses representing myogenic and endothelial regulation, respectively. This computational framework is applied to large, semirealistic microvascular networks derived from in vivo mouse data, enabling single-vessel resolution under both physiological and pathological conditions.

An important unresolved question is whether autoregulation is achieved through uniform vascular adjustments across the arterial network or through hierarchical patterns of responsiveness among different vascular compartments. If such a hierarchy exists, how do vessel diameter or spatial location within the network impact the responsiveness of individual vessels? Such analyses are particularly important given the substantial interindividual variability in vascular network anatomy, which may influence how autoregulatory responses are distributed throughout the cerebrovascular network. Our framework further enables the quantification of how structural differences across brain regions, such as penetrating arterial tree density, affect the network’s ability to maintain stable perfusion.

Quantifying these spatial differences in cerebral autoregulation is particularly relevant in pathological states in which vascular tone regulation becomes disrupted. In such conditions, arteries no longer actively respond to changes in pressure but instead behave passively, leading to potentially harmful variations in cerebral perfusion even with small blood pressure fluctuations. Autoregulation dysfunction has been implicated in several pathological conditions, including ischemic stroke (18–20), traumatic brain injury (21), orthostatic hypotension (22), and neurodegenerative diseases such as Alzheimer’s disease (23, 24). In the context of ischemic stroke, typically caused by a blood clot obstructing a major cerebral artery (25), experimental studies have focused on arteries within the affected region. One key finding suggests that during ischemia followed by reperfusion, these arteries lose their ability to constrict in response to elevated intravascular pressure, a hallmark of disrupted myogenic response (14, 26).

Clinical studies have examined changes in static and dynamic cerebral autoregulation across the acute (<48 h), subacute (48 h to 7 d), and chronic (>7 d) phases of ischemic stroke (19). However, given the risks associated with blood pressure manipulations in the acute phase, clinical data on static autoregulation in these conditions are limited (19, 27–31). Variability in study design (e.g., timing of assessment or stroke subtype) likely explains the mixed findings reported in the literature, especially during the acute phase (19, 28, 30). Evidence pointing to impaired autoregulation in the subacute phase is more robust, and partial or complete recovery during the chronic phase is reported for most patients, except for cases with particularly severe strokes (19, 28). Despite its clinical importance, the contribution of individual vessels to chronic autoregulatory dysfunction and how it affects perfusion remains largely unexamined, hindering the development of novel therapies to restore vasoreactivity and improve patient outcomes.

Interestingly, during the acute phase, alterations in autoregulatory mechanisms might cause different reperfusion dynamics and thereby contribute to determining the poststroke outcome (26, 29, 32–35). Recent in vivo studies have shown that reperfusion profiles following middle cerebral artery (MCA) occlusion tend to correlate with the extent of leptomeningeal collaterals (LMCs) (36, 37). LMCs are anastomotic connections between major cerebral arteries that provide alternative pathways for blood flow to sustain perfusion to underperfused regions during arterial occlusion. Their extent varies widely among individuals, and richer collateral networks are generally associated with reduced infarct volumes and better clinical outcomes (25, 38, 39). In mice, rapid and excessive hyperemic reperfusion has been observed for strains with poor collateralization, whereas those with abundant LMCs undergo a more gradual and controlled restoration of flow (36). These contrasting reperfusion profiles could be caused by ischemia-induced endothelial damage, which impairs the vasoregulatory capacity of the recanalized MCA segment. In contrast, rich collateral networks may sustain minimal perfusion during occlusion, preserving endothelial function and enabling more physiologically regulated reperfusion upon recanalization (36). However, this hypothesis has not yet been systematically tested. Particularly, it remains unclear how the extent of LMCs influences perfusion in the proximal MCA territory during ischemic stroke and whether collateralization serves as the principal determinant of these divergent reperfusion responses.

Computational models offer a powerful tool, enabling the controlled investigation of individual factors and their influence on cerebral hemodynamics. Over the past decades, various computational models have been developed to describe both static and dynamic cerebral autoregulation (12, 40–47). Most of these employ a compartmental approach, in which vascular territories or vessel segments are simplified and grouped into lumped compartments. While this offers computational efficiency and yields insights into global autoregulatory behavior, it drastically simplifies the structural and functional heterogeneity of the microvasculature (48, 49) and does not allow investigation of mechanisms at a single-vessel resolution. As such, these models cannot account for the spatial heterogeneity and vessel-specific regulatory responses that are critical for understanding blood flow regulation at the microvascular level. Recent advances have attempted to overcome these limitations. Daher et al. (50) introduced a network-based modeling approach for dynamic cerebral autoregulation, describing the myogenic and endothelial responses. Additionally, Esfandi et al. (51) developed a static autoregulation model using a constriction transfer function that links rising pressure to myogenic vasoconstriction, applied to a simplified vascular network.

Here, we introduce a model of static cerebral autoregulation that operates at the level of individual arteries within large, semirealistic microvascular networks and incorporates both myogenic and endothelial control mechanisms. Building on the dynamic autoregulation framework of Daher et al. (50), we adapted the model to investigate how changes in mean arterial pressure (MAP) are redistributed across vascular networks and how the density of penetrating arterial trees influences autoregulatory capacity. We further examined how chronic autoregulatory dysfunction after stroke alters pressure–flow relationships and capillary perfusion, and whether impaired regulation can explain the hyperperfusion patterns observed during reperfusion in vivo (36).

## Materials and Methods

### Microvascular Networks.

**Fig. 1.**
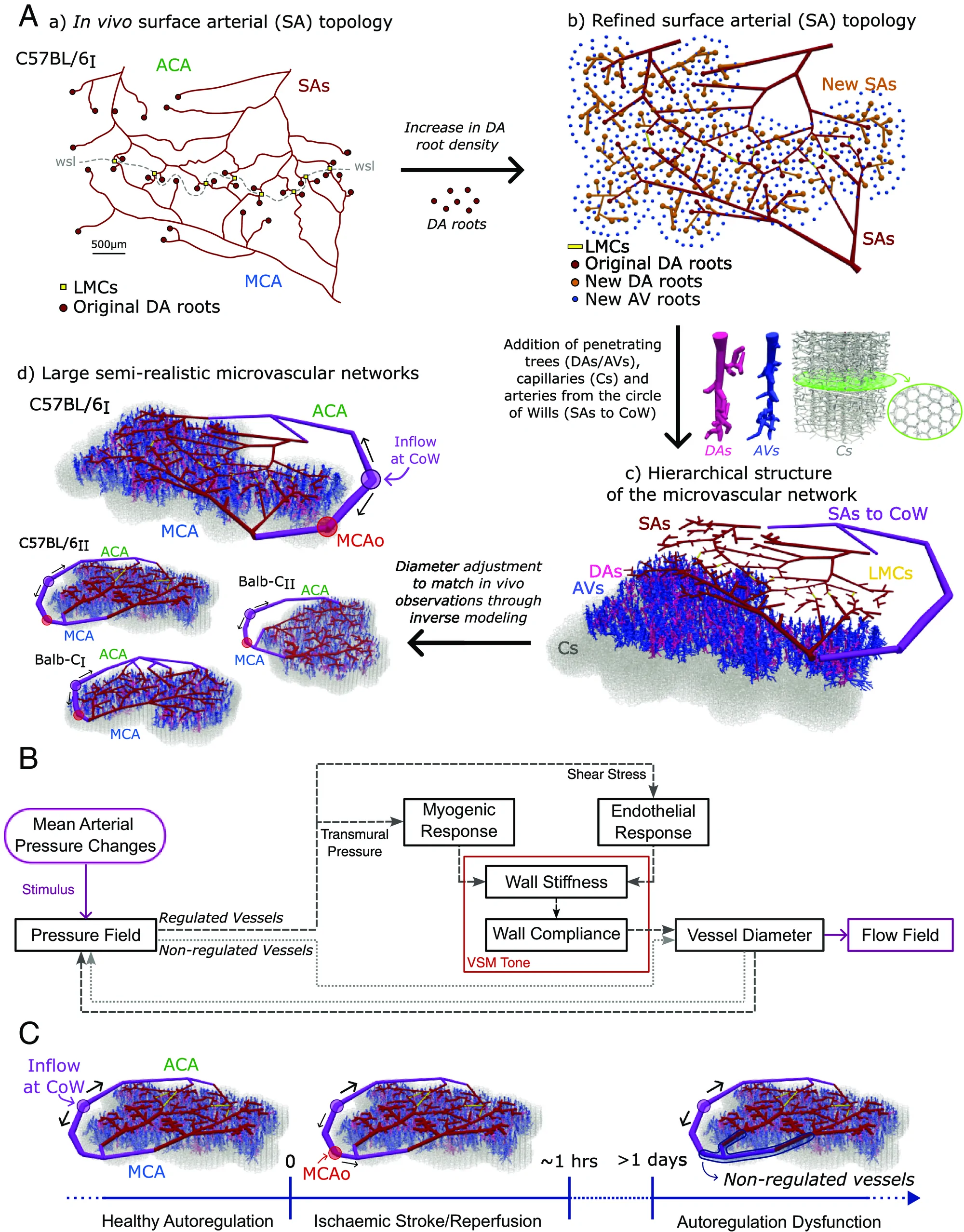
Semirealistic microvascular networks and overview of the autoregulation model. (*A*) Stepwise generation of semirealistic microvascular networks. (*a*) In vivo surface arterial (SA) topology of the C57BL/6_I_ mouse showing middle cerebral artery (MCA) and anterior cerebral artery (ACA) branches connected by leptomeningeal collaterals (LMCs, yellow squares) across the watershed line (wsl); original DA root points are shown in red. (*b*) Refined SA topology with increased DA root density (orange circles), ascending vein (AV) roots (blue circles), and newly added SAs (orange lines). (*c*) Hierarchical network structure including DAs, AVs, and an artificial capillary bed. Additional SAs connect MCA and ACA territories to a common inflow representing the Circle of Willis (CoW). (*d*) Semirealistic microvascular networks generated from two C57BL/6 and Balb-C mice (each denoted by subscripts I and II), showing inflow locations (purple circles) and MCA occlusion (MCAo) sites (red circles). (*B*) Schematic representation of the autoregulation model. The flow chart for regulated and nonregulated vessels is depicted by dashed and dotted lines, respectively. (*C*) Schematic timeline of simulated autoregulatory conditions including (i) healthy conditions, (ii) ischemic stroke followed by reperfusion (~1 h), and (iii) chronic autoregulation dysfunction (>1-d postocclusion).

Simulations were performed on four previously generated semirealistic microvascular networks (MVNs) derived from two mouse strains (C57BL/6 and Balb-C) with different collateral extent and descending arteriole (DA) density (52, 53). Fig. 1*A* summarizes the network generation workflow, starting from in vivo surface arterial (SA) networks. To ensure agreement with experimentally observed hemodynamics, vessel diameters were calibrated using an inverse modeling approach based on in vivo red blood cell velocity measurements (52). Additional details are provided in the *SI Appendix*.

### Static Cerebral Autoregulation Model.

Blood flow was computed using a graph-based approach where vessels are modeled as edges governed by Poiseuille’s law, assuming laminar, quasi-steady flow (48, 54–59). Relative viscosity was calculated using the in vitro model by Pries et al. (60) and pressure boundary conditions were applied at a single arterial inflow and venous outlets. A detailed description of the flow model, equations, and assumptions is provided in the *SI Appendix*.

We modeled blood vessels as elastic tubes whose diameters adapt to changes in pressure either through pressure-driven elastic deformation or through active autoregulatory mechanisms. When vessels respond solely through elastic deformation, they dilate with rising pressure or collapse with falling pressure, leading to pressure-dependent changes in CBF. Active behavior arises from cerebral autoregulatory mechanisms, which adjust VSM tone to maintain stable CBF. These active responses allow regulatory vessels to constrict or dilate to counteract pressure changes and preserve constant CBF. In our model, we distinguish between nonregulated vessels (ascending venules, AVs, and capillaries) and regulated vessels (all arteries, i.e., surface artery-to-Circle of Willis vessels (SAs-to-CoW), SAs, and DAs).

The nonregulated vessel response is described by a pressure–area relationship derived from linear elastic theory (neglecting viscoelastic effects), that describes how transmural pressure alters the vessel cross-sectional area (61). We employed a widely used nonlinear formulation (62):

[1] $$p_{t,ij}=\beta_{\mathrm{ij}}\left(\sqrt{A_{\mathrm{ij}}}-\sqrt{A_{ref,ij}}\right),$$

where $p_{t,ij}=p_{\mathrm{ij}}-p_{ext,ij}$ is the transmural pressure of each vessel, computed as the difference between the intraluminal $p_{\mathrm{ij}}$ and extravascular pressure $p_{ext,ij}$. The intraluminal pressure is $p_{\mathrm{ij}}=0.5\left(p_{i}+p_{j}\right)$ and $p_{ext,ij}=0mmHg$ is assumed (41, 50, 61). A sensitivity analysis for physiological variations in extravascular pressure is provided in *SI Appendix*, Fig. S1. $A_{\mathrm{ij}}$ is the current cross-section of the vessel and $A_{ref,ij}$ indicates the reference cross-section at zero transmural pressure. $A_{ref,ij}$ and $r_{ref,ij}$, (reference radius) of all vessels were calculated, using the diameter and pressure field at baseline conditions and based on the linear elastic theory:

[2] $$r_{ref,ij}=\frac{E_{\mathrm{ij}}h_{\mathrm{ij}}r_{\mathrm{ij}}}{\left(1-v^{2}\right)p_{t,ij}r_{\mathrm{ij}}+E_{\mathrm{ij}}h_{\mathrm{ij}}},$$

where $r_{\mathrm{ij}}$ is the vessel radius, $E_{\mathrm{ij}}$ the Young’s Modulus, $h_{\mathrm{ij}}$ the vessel wall thickness. v is the Poisson’s ratio and v=0.5 assuming that the vessel wall material is incompressible. The proportionality coefficient $\beta_{\mathrm{ij}}$ determines the elastic relationship between transmural pressure and vessel cross-sectional area in Eq. **1** and is defined as ref. 62:

[3] $$\beta_{\mathrm{ij}}=\frac{4}{3}\frac{\sqrt{\pi }E_{\mathrm{ij}}\;h_{\mathrm{ij}}}{A_{ref,ij}}.$$

Therefore, for nonregulated vessels, diameters $d_{\mathrm{ij}}$ were calculated by combining Eqs. **1** and **3**:

[4] $$d_{\mathrm{ij}}=d_{ref,ij}\left(1+\frac{3}{8}\frac{d_{ref,ij}}{E_{\mathrm{ij}}\;h_{\mathrm{ij}}}p_{t,ij}\right).$$

In contrast, regulated vessels can actively adjust vessel wall compliance through autoregulatory mechanisms, thereby modifying the pressure–area relationship (11, 50). To capture this behavior, Eq. **1** is actively modulated by controlling the level of VSM tone through changes in compliance. Compliance $C_{\mathrm{ij}}$ is defined as the change in arterial volume $V_{\mathrm{ij}}$ relative to the change in pressure $p_{t,ij}$ (50):

[5] $$C_{\mathrm{ij}}=\frac{{\mathrm{dV}}_{\mathrm{ij}}}{{\mathrm{dp}}_{t,ij}}.$$

Applying Eq. **5** to the pressure–area relationship in Eq. **1** and assuming a constant area along the vessel such that $V_{\mathrm{ij}}=L_{\mathrm{ij}}A_{\mathrm{ij}}$, compliance $C_{\mathrm{ij}}$ can be also expressed in terms of $\beta_{\mathrm{ij}}$:

[6] $$C_{\mathrm{ij}}={\left(\frac{\partial p_{t}\left(V\right)}{\partial V}\right)}^{-1}=\frac{2\sqrt{L_{\mathrm{ij}}\;V_{\mathrm{ij}}}}{\beta_{\mathrm{ij}}}.$$

Accordingly, baseline compliance $C_{o,ij}$ can be written as a function of the baseline radius $r_{o,ij}$ and reference radius $r_{ref,ij}$:

[7] $$C_{o,ij}=\frac{3}{2}\frac{\pi L_{\mathrm{ij}}\;r_{o,ij}\;r_{ref,ij}^{2}}{E_{\mathrm{ij}}\;h_{\mathrm{ij}}}.$$

To model active regulation, we incorporated a quasi-steady feedback model that captures myogenic and endothelial responses. Following Daher et al. (50), the relative vessel wall stiffness $k_{\mathrm{ij}}$ is given by

[8] $$k_{\mathrm{ij}}=1+S_{\sigma ,ij}\left(\frac{p_{t,ij}}{p_{to,ij}}-1\right)-S_{\tau ,ij}\left(\left|\frac{\tau_{\mathrm{ij}}}{\tau_{o,ij}}\right|-1\right),$$

where $S_{\sigma ,ij}$ and $S_{\tau ,ij}$ are sensitivity factors for myogenic and endothelial mechanisms, respectively. $S_{\sigma ,ij}$ is larger than $S_{\tau ,ij}$ because the myogenic mechanism is typically the dominant regulatory process (43, 50). The myogenic response is driven by changes in transmural pressure $p_{t,ij}$, relative to its baseline value $p_{to,ij}$. The endothelial response depends on changes in wall shear stress $\tau_{\mathrm{ij}}$, compared to its baseline value $\tau_{o,ij}$. The baseline values, $p_{to,ij}$ and $\tau_{o,ij}$ are obtained from the baseline flow and pressure fields. Additionally, aligned with in vivo studies (63–65), a threshold of $\tau_{\mathrm{ij}}$ = 0.1 Pa was enforced to ensure that only physiologically relevant levels of shear stress elicit a regulatory response. For vessels where the average $\bar{\tau_{\mathrm{ij}}}$ over the first 20 iterations was <0.1 Pa the endothelial response was set to 0. The wall shear stress, $\tau_{\mathrm{ij}}$, in each vessel was calculated assuming a parabolic velocity profile typical for laminar pipe flow, Eq. **9**. The impact of alternative velocity profile assumptions is assessed in the *SI Appendix*.

[9] $$\tau_{\mathrm{ij}}=\frac{32\;q_{\mathrm{ij}}\;\mu_{p}\;\mu_{rel,ij}}{\pi \;d_{\mathrm{ij}}^{3}}.$$

As compliance can only vary within set limits from its baseline value, a sigmoidal function links the stiffness $k_{\mathrm{ij}}$ to relative compliance $C_{r,ij}$ (40, 42, 50):

[10] $$\begin{array}{c} C_{\mathrm{ij}}=C_{0,ij}\;C_{r,ij}=C_{0,ij}\left[1+{\left(\Delta C^{\pm }/C_{0}\right)}_{\mathrm{ij}}\text{tanh}\left(\frac{1}{G_{\mathrm{ij}}}\frac{1}{{\left(\Delta C^{\pm }/C_{0}\right)}_{\mathrm{ij}}}\left(\frac{1}{k_{\mathrm{ij}}}-1\right)\right)\right], \end{array}$$

where ${\left({\Delta C}^{\pm }/C_{0}\right)}_{\mathrm{ij}}$ defines the bounds for positive and negative compliance changes (asymmetrically constrained) and $G_{\mathrm{ij}}$ sets the steepness of the sigmoid slope. The values of parameters $\left(S_{\sigma ,ij},\;S_{\tau ,ij},\;G_{\mathrm{ij}},\;{\left(\Delta C^{\pm }/C_{0}\right)}_{\mathrm{ij}}\right)$ were selected based on values reported in prior computational studies (50) (*SI Appendix*, Table S1).

By computing the compliance according to Eqs. **8** and **10** and subsequently updating parameter $\beta_{\mathrm{ij}}$ according to Eq. **6**, changes in compliance directly modify the pressure–area relationship and allow the vascular system to adapt to pressure changes and to reach a new equilibrium. By substituting Eqs. **1**, **6**, and **10**, the diameter $d_{\mathrm{ij}}$ of regulatory vessels under active control can be computed as

[11] $$d_{\mathrm{ij}}=0.5\;d_{ref,ij}\left(1+\sqrt{1+3\frac{r_{o,ij}\;C_{r,ij}}{E_{\mathrm{ij}}\;h_{\mathrm{ij}}}p_{t,ij}}\right).$$

The minimal achievable diameter per vessel corresponds to the reference diameter $d_{\mathrm{ref}}$ (Eqs. **4** and **11**), occurring when the transmural pressure $p_{t}$ approaches zero. This constraint is inherent to the model definition, but in agreement with in vivo observations (5).

As pressure and diameter are mutually dependent, the system needs to be solved iteratively. At each iteration, pressure and flow rates are computed using the blood flow model. Based on the resulting pressure and flow field the transmural pressure and shear stress is evaluated to determine the relative stiffness of regulatory vessels (Eq. **8**), update vascular compliance (Eq. **10**) and subsequently compute new vessel diameters (Eq. **11**). For the nonregulated vessels, diameter changes are solely driven by pressure variations (Eq. **4**). The iterative loop is repeated until convergence, defined as a maximum relative change in diameter below 10^−6^, was reached (Fig. 1*B*). This was typically achieved within 150 iterations, using a relaxation factor of 0.1 (0.01 for reperfusion simulations). All parameter values used in our in silico model are provided in *SI Appendix*, Table S1. A sensitivity analysis of key autoregulatory model parameters is summarized in the *SI Appendix*.

## Results

We analyzed cerebral autoregulatory behavior under three conditions: i) healthy, ii) stroke-reperfusion, and iii) chronic autoregulation dysfunction conditions (Fig. 1*C*). Within each setup, we investigated specific relevant aspects of autoregulatory control and vascular adaptation.

### Vascular Responses Are Heterogeneous and Vary with Arterial Location.

**Fig. 2.**
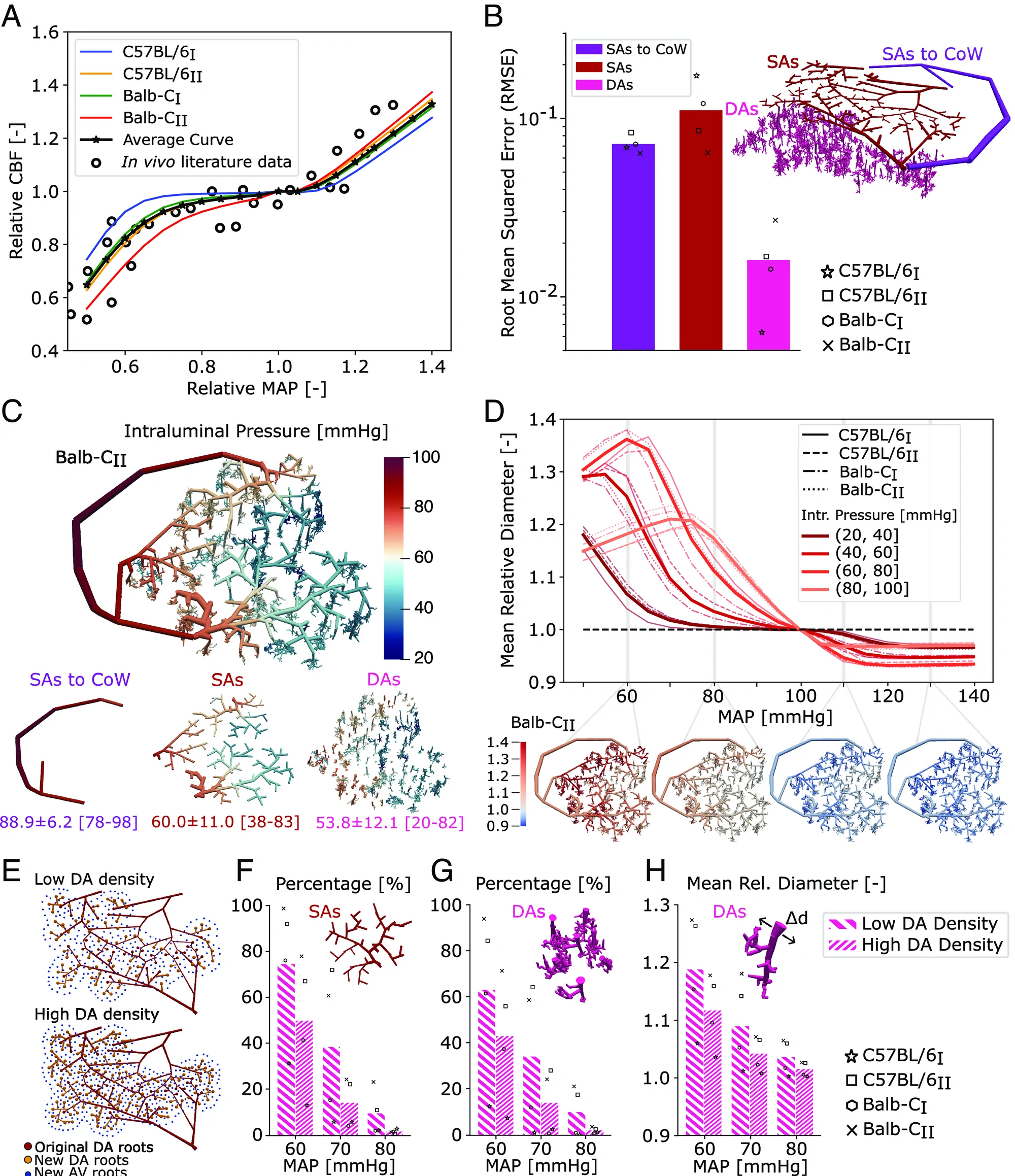
The role of different arteriole levels for cerebral autoregulation under healthy conditions. (*A*) Static autoregulation curves for all microvascular networks compared to in vivo experimental measurements (66). (*B*) Root Mean Squared Error (RMSE) between the fully regulated and the modified autoregulation curve, computed by deactivating different arterial groups, namely: (i) surface arteries connecting to the circle of Willis (SAs-to-CoW), (ii) surface arteries (SAs), and (iii) descending arterioles (DAs). Bars represent mean across four networks. (*C*) Intraluminal pressure distribution across all arterial segments in the Balb-C_II_ microvascular network at baseline conditions. Summary statistics (mean ± SD and range) are reported for each arterial group. (*D*) Mean relative diameter change as a function of MAP across four baseline intraluminal pressure intervals: (20, 40], (40, 60], (60, 80], and (80, 100] mmHg. The nonsolid lines show the averaged relative diameter change across all vessels within each pressure group per network and the solid line the average across networks. The *Bottom* panel shows spatial maps of relative diameter changes across the Balb-C_II_ network at selected MAP values. (*E*) Two surface SA topologies derived from C57BL/6_I_ mouse illustrating low and high DA root densities, respectively. (*F* and *G*) Proportion of vessels showing >10% dilation in response to MAP drops (60, 70, 80 mmHg) across different DA densities: (*F*) SAs, and (*G*) DA trees (mean dilation per tree). (*H*) Mean relative diameter across all DA trees at 60, 70, and 80 mmHg, comparing low and high DA density cases. For each DA tree, the mean relative diameter was computed, followed by averaging across all trees in the network.

To mimic healthy autoregulation, we started from the baseline state of each network and varied the MAP (inflow pressure) within the range of 50 to 140 mmHg, while maintaining active diameter regulation in all arterial segments. First, we validated our static autoregulation model by reconstructing the classic static autoregulation curve (Fig. 2*A*). Based on the average curve, the autoregulatory range, i.e., the MAP interval where CBF remains within ±10% of its baseline value, was 68 – 118 mmHg, consistent with experimental observations (15, 66).

To quantify the role of different arterial groups in maintaining CBF, we selectively deactivated the autoregulatory ability in three arterial groups independently, namely: i) SAs-to-CoW, ii) SAs, and iii) DAs (Fig. 2*B*). In each case, the affected arteries behaved as nonregulated vessels and responded solely through the elastic pressure–area relationship to changes in MAP, after which a modified autoregulation curve was calculated. The root mean squared error (RMSE) relative to the fully regulated curve (control) was smallest when autoregulation was deactivated in DAs, suggesting that SAs-to-CoW and SAs were able to compensate for the absence of DA vasodynamics (Fig. 2*B*). In contrast, deactivating vasodynamics in SAs-to-CoW or SAs substantially changes the autoregulatory curve (*SI Appendix*, Fig. S2).

To further investigate how individual arteries contribute to MAP changes, we spatially quantified the relative diameter changes in terms of intraluminal pressure (IP), which serves as a proxy for the arterial location within the network (Fig. 2*C*). The most upstream arteries (IP: 80 to 100 mmHg) responded to smaller MAP variations, reaching maximum dilation for an MAP of ~75 mmHg (Fig. 2*D*). In contrast, more distal arteries (IP: 60 to 80 mmHg) only reacted when the pressure drop became more pronounced, reaching their peak dilation at ~60 mmHg and exceeding the dilation amplitude observed in the most upstream vessels (*SI Appendix*, Fig. S3*A*). Pressure perturbations were distributed nonuniformly across the arterial tree, with proximal arteries affected first and distal arteries only at larger MAP reductions, consistent with sequential autoregulatory recruitment (*SI Appendix*, Fig. S4).

During hypertension, arterial diameters decreased by less than 10% relative to baseline and remained near this constricted state with further MAP increases (*SI Appendix*, Fig. S3*B*). Arteries located closer to the CoW (IP: 90 to 100 mmHg) exhibited their strongest constriction following a 10% increase in MAP and remained constricted with subsequent MAP elevations. Arteries with baseline intraluminal pressures between 70 to 80 mmHg showed the largest diameter reductions across all levels of MAP increase (*SI Appendix*, Fig. S3*B*). Analysis of arterial diameter responses grouped according to the Young’s modulus classes used in the model demonstrated stronger size-dependent vasodilatory behavior, whereas vasoconstrictive responses remained comparatively similar across vessel groups (*SI Appendix*, Fig. S5). This behavior is qualitatively consistent with experimental observations of cerebral autoregulation (4, 5, 17). Comparison with representative in vivo and ex vivo pressure–diameter measurements further showed that the model reproduces the principal autoregulatory trends observed experimentally across vessel sizes (*SI Appendix*, Fig. S6). In particular, experimental studies have reported relatively modest pial arteriolar constriction (typically >92% of baseline diameter) during hypertension, whereas vasodilatory responses during hypotension were substantially larger and more size dependent (5, 17). The simulated responses exhibited the same overall behavior, including stronger vasodilation in smaller vessels and comparatively modest vasoconstriction at elevated pressures.

### Descending Artery Density Increases Vascular Autoregulatory Capacity.

In agreement with in vivo observations, our microvascular networks differed in DA density. To assess how this anatomical feature influences the autoregulatory response, we adjusted the DA density in each of the four original networks to generate high- and low-density variants, matching literature values for C57BL/6 (13.4 DAs/mm^2^) and Balb-C (8.9 DAs/mm^2^), respectively (52) (Fig. 2*E*). Importantly, LMC characteristics were maintained, and only DA root density was modified (step b, in Fig. 1*A* and *SI Appendix*, Table S2).

Although the overall shape of the autoregulation curve was generally maintained for networks with different DA densities, we observed more extensive arterial dilation in the networks with lower DA density (*SI Appendix*, Fig. S7). Networks with fewer DAs exhibited a higher proportion of SAs and DA trees dilating by at least 10% in response to a MAP decrease (Fig. 2 *F* and *G*). For example, in networks with fewer DAs, twice as many SAs dilated by at least 10% following a 30% MAP reduction (70 mmHg) compared to networks with higher DA density (38.5% vs. 14.1%, Fig. 2*F*). Additionally, DAs exhibited more pronounced dilation in low-DA-density networks, with a 40% decrease in MAP (60 mmHg) leading to 18.8% dilation, compared to 11.7% in high-DA-density networks (Fig. 2*H*). In summary, these results suggest that networks with a low DA density might be more susceptible to autoregulatory impairments, while networks with a high DA density are characterized by a higher autoregulatory capacity.

### Autoregulatory Failure Leads to Capillary Flow Maldistribution Across MAP Variations.

Next, we sought to assess how autoregulatory failure affects capillary perfusion. Following ischemic stroke, autoregulation may become impaired, resulting in unadjusted CBF responses to pressure variations. As vessels on the MCA side downstream of the previously occluded segment are most likely to be affected, we considered two impairment strategies: i) *upstream-to-downstream:* starting from larger upstream SAs and progressively including smaller downstream arteries, and ii) *downstream-to-upstream:* beginning with the most distal SAs and extending the loss of regulation toward more proximal vessels. In both strategies, the DAs extending from the impaired SAs were also assumed to be affected.

The score of autoregulatory impairment was quantified as the RMSE between the original (fully regulated) autoregulation curve and the progressively impaired curve. This RMSE was then normalized by the RMSE between the fully regulated curve and the fully impaired case, in which all arteries on the MCA side downstream of the previously occluded segment were assumed to have lost their autoregulatory function (i.e., nonregulated vessels, *SI Appendix*, Fig. S8*A*). An impairment score of 100% indicates that the impaired autoregulation curve is indistinguishable from the fully impaired case. The original, ~55% impaired and 100% impaired curves are illustrated in *SI Appendix*, Fig. S8*B*.

**Fig. 3.**
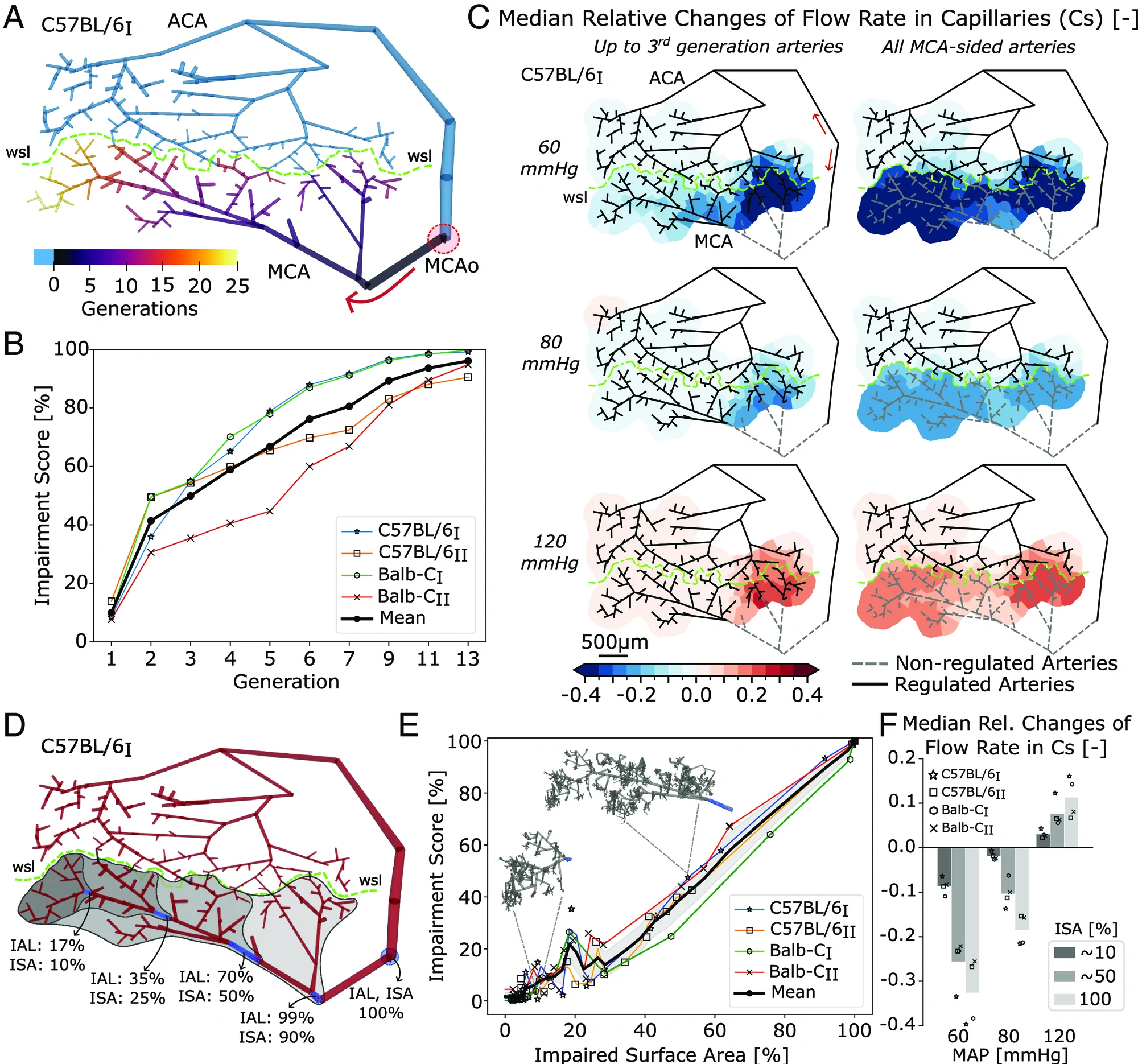
Stroke-induced autoregulatory impairment based on generation- and area-specific analysis. (*A*) Arterial generation map for the C57BL/6_I_ network, used to implement the *upstream-to-downstream* impairment approach. First-generation arteries are defined as those immediately downstream of the previously occluded segment (marked by the red dashed circle), with each subsequent generation defined by successive bifurcations. (*B*) Autoregulation impairment score as a function of the number of impaired arterial generations. Results are shown for each network and averaged across networks. (*C*) Spatial maps of median relative changes in capillary flow rate at three mean arterial pressure (MAP) levels (60, 80, and 120 mmHg), for two impairment scenarios: (i) impairment restricted to surface arteries up to the 3rd generation (*Left*), and (ii) impairment of all arteries on the MCA side (*Right*). Regulated and nonregulated arteries are indicated with dashed and solid lines, respectively. The relative change is computed with respect to the fully regulated case at each pressure level. (*D*) Surface topology of the C57BL/6_I_ network showing progressive impairment of surface arteries, used in the *downstream-to-upstream* impairment approach. Impairment is quantified in terms of impaired arterial length (IAL) and impaired surface area (ISA), both expressed as percentages. IAL is the ratio of impaired arterial length to total MCA-sided arterial length; ISA is the normalized 2D convex hull area enclosing the impaired surface arteries. The location with 100% IAL and ISA corresponds to the site of the original occlusion. (*E*) Autoregulation impairment score as a function of impaired surface area. Results are shown for each network and averaged across networks. *Insets* illustrate representative surface artery configurations corresponding to different ISA levels. (*F*) Median relative changes in capillary (Cs) flow rate at 60, 80, and 120 mmHg for three levels of impairment: ~10%, ~50%, and 100% impaired surface area. Capillaries were selected within 300 μm of impaired SAs. The relative change is computed with respect to the fully regulated case at each pressure level. Bars represent mean across four networks.

For the upstream-to-downstream strategy, MCA-sided surface arteries (SAs-to-CoW and SAs) downstream of the occluded vessel are divided into generations, where each generation represents a new bifurcation (Fig. 3*A*). Impairing arteries up to the third generation resulted in an autoregulation impairment score of ~50%, indicating that these arteries play a key role in regulating blood flow in response to pressure variations (Fig. 3*B*). This suggests that loss of vasoreactivity in the first major artery downstream of the occlusion can substantially impair autoregulation even after recanalization. To evaluate how this impairment spatially affects perfusion at the capillary level, we computed the median relative change in flow rate for capillary columns positioned below DA root points. We considered three MAP values, i.e., 60, 80, and 120 mmHg, and two impairment scenarios: zero-to-third-generation impairment and complete MCA-sided impairment (Fig. 3*C*). At 60 mmHg, capillary flow decreased markedly, with relative flow change reaching −42% under full MCA impairment and −13% under third-generation impairment. At 80 mmHg, the reduction was smaller (−20% under full MCA impairment), whereas at 120 mmHg, loss of autoregulation resulted in hyperperfusion, reaching +16%.

To assess a complementary impairment scenario, we assumed that vessels experiencing more severe hypoperfusion are more susceptible to losing their autoregulatory capacity. Because these vessels are typically located in distal regions and exhibit lower perfusion under both baseline and stroke conditions (*SI Appendix*, Fig. S9), impairment was progressively extended from distal to proximal arteries (Fig. 3*D*). For each region, we quantified the percentage of impaired artery length (IAL) and impaired surface area (ISA), reflecting the cumulative extent of impairment. When ~45 to 50% of the distal arterial surface area was impaired, the autoregulatory impairment score reached ~40% (Fig. 3*E*). Using the same capillary flow analysis, full MCA impairment reduced capillary flow by ~34% at 60 mmHg and produced hyperperfusion of ~12% at 120 mmHg (Fig. 3*F*). Spatial maps for different impairment scenarios are shown in *SI Appendix*, Fig. S10.

Overall, these results demonstrate that the spatial impact of autoregulatory failure depends on both MAP deviation and the extent of arterial impairment. While localized impairment leads to moderate region-specific effects, the loss of regulation in all MCA-sided arteries results in widespread perfusion disturbances, with changes propagating across the entire microvascular network. This analysis highlights the crucial role of proximal surface arteries in maintaining microvascular perfusion under both hypotensive and hypertensive conditions.

### Altered Vasodynamics Are a Plausible Cause for CBF Overshoot During Reperfusion.

As previously described, in vivo experiments have shown that reperfusion dynamics following stroke may be impacted by the extent of LMC (36). To isolate the effect of LMCs, we artificially modified the number of LMCs in each of our four original networks (derived from two C57BL/6 and two Balb-C mice), creating two configurations per network: one with abundant collaterals (100% LMCs) and one without collaterals (0% LMCs, see *SI Appendix* for details). All paired configurations differ only in the number of LMCs and are otherwise identical.

To mimic MCA occlusion (MCAo), a 50 μm segment of the MCA-sided SAs-to-CoW (M2 segment) artery was constricted to 10% of its baseline diameter. MAP was kept constant at 100 mmHg, and vessel diameters were adjusted in response to the occlusion (i.e., stimulus: MCAo). To obtain representative diameter adjustments under stroke conditions and to isolate the role of autoregulatory mechanisms in shaping reperfusion dynamics, we adopted a computational strategy in which all vessels, except LMCs, were treated as nonregulated during stroke and actively regulated during reperfusion. This assumption reflects the ischemic environment, in which perfusion pressures fall below the typical autoregulatory range, resulting in predominantly pressure-driven elastic vessel responses. Moreover, in vivo studies suggest that under stroke conditions, pial vessels tend to either reduce their diameter (67) or show no significant diameter changes (68), further supporting our model assumption of nonregulated vessel behavior during MCAo. Reintroducing active regulation during reperfusion enables a controlled evaluation of how vessel responsiveness modulates flow restoration. LMCs retained active autoregulation throughout both the stroke and reperfusion phases, consistent with experimental evidence showing their vasodilatory behavior during ischemia (36, 47, 67).

**Fig. 4.**
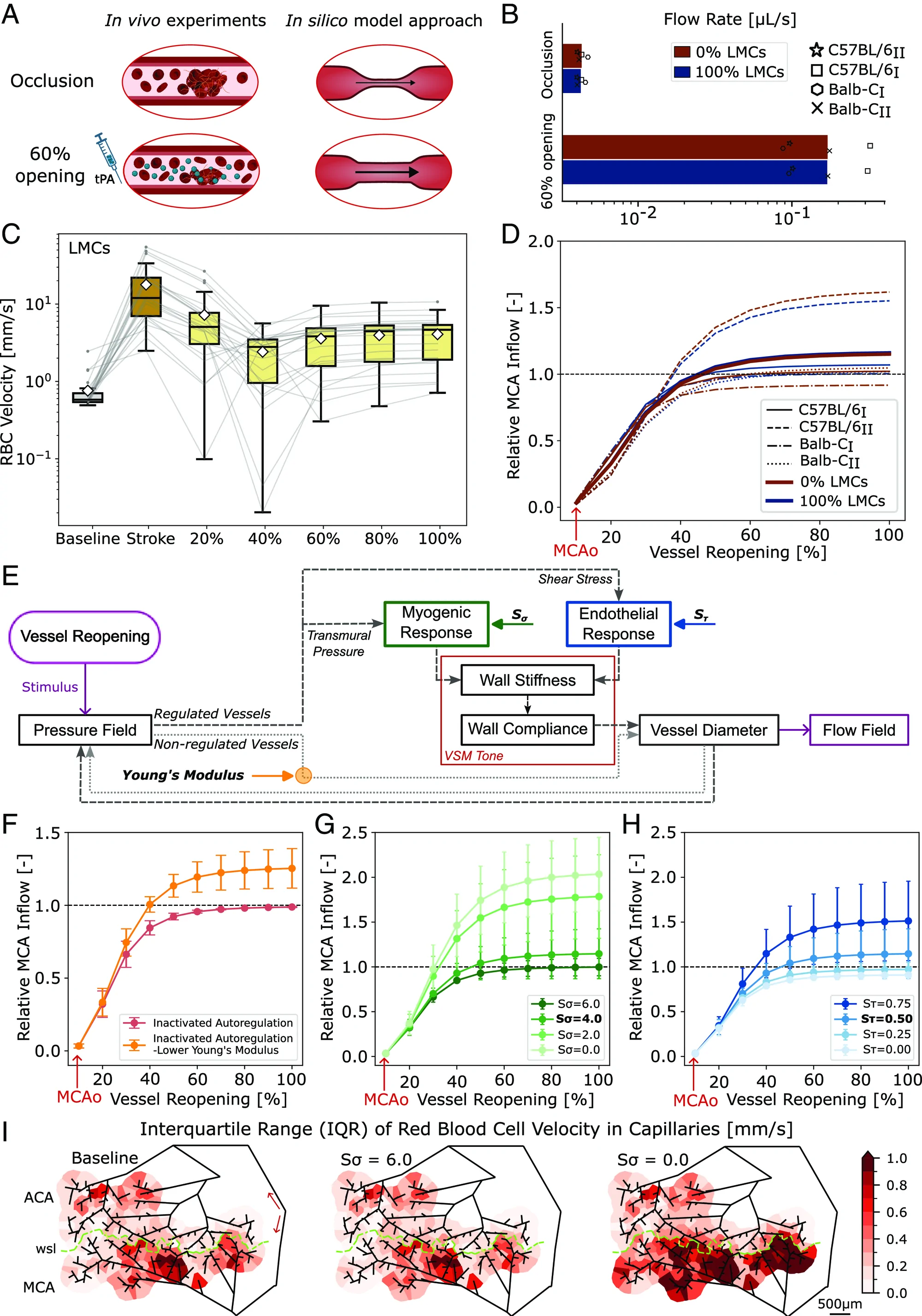
The role of vascular topology and reactivity in reperfusion after MCA occlusion. (*A*) Schematic of experimental and computational models of vessel reopening after stroke. (*Left*) In vivo MCA occlusion and exemplary partial reopening following tPA treatment. (*Right*) In silico simulation of controlled vessel reopening to a defined percentage of baseline diameter. (*B*) Flow rate [μL/s] through the occluded vessel during stroke (vessel diameter at 10% of baseline) and reperfusion (vessel reopened to 60% of baseline) conditions for 0% and 100% leptomeningeal collaterals (LMCs). Bars represent mean across all networks. (*C*) RBC velocity [mm/s] through all LMCs across all microvascular networks (MVNs) under baseline, stroke, and reopening conditions. (*D*) Relative MCA inflow as a function of vessel reopening, shown for each network with distinct line styles and for both 0% and 100% LMC conditions. Bold lines indicate the average across all networks for each condition. (*E*) Schematic of the autoregulatory model used to model reperfusion, highlighting mechanisms potentially affected by vascular impairments in response to stroke. Independently altered characteristics are myogenic ($S_{\sigma }$) and endothelial ($S_{\tau }$) responses in regulated vessels, and Young’s modulus in a subset of vessels when autoregulation was deactivated and vessel diameters responded solely through the elastic pressure–area relationship. (*F*–*H*) Relative MCA inflow as a function of vessel reopening under altered regulatory mechanisms: (*F*) the absence of active autoregulation, with and without reduced Young’s modulus; (*G*) active autoregulation with varying myogenic sensitivity ($S_{\sigma }$ = 0, 2, 4, 6); (*H*) active autoregulation with varying endothelial sensitivity ($S_{\tau }$ = 0, 0.25, 0.5, 0.75). All curves show mean ± SD across networks. Baseline sensitivity factors are highlighted in bold. (*I*) Spatial distribution of the interquartile range (IQR) of red blood cell (RBC) velocities [mm/s] in capillaries under baseline (prestroke) conditions and after full reopening of the previously occluded vessel, shown for strong ($S_{\sigma }$ = 6.0) and absent ($S_{\sigma }$ = 0.0) myogenic response.

Reperfusion occurs due to tissue plasminogen activator (tPA) treatment, which was modeled by stepwise reopening of the occluded vessel (Fig. 4*A*). At this stage, the stroke networks served as the baseline case, i.e., all baseline variables (denoted with subscript *o*) have been updated accordingly. MAP is held constant at 100 mmHg throughout the gradual reopening. Vessel diameters now change in response to pressure redistributions caused by the reopening of the occluded segment (i.e., stimulus: gradual vessel reopening).

In response to MCAo, all arteries, except LMCs, underwent constriction of less than 10% from their baseline diameter, while LMCs dilated by approximately 23%, consistent with experimental observations (36, 67) (*SI Appendix*, Table S3). To investigate the role of LMCs during MCAo and reperfusion, we assessed perfusion in the occluded vessel for different network configurations (100% and 0% LMCs) under *stroke* and *reperfusion conditions*. No substantial difference in perfusion in the occluded vessel itself was found for networks with 100% and 0% LMCs for stroke and 60% reopening (Fig. 4*B*). Thus, LMC density seemingly does not significantly affect perfusion in the recanalized segment, despite its critical role in regions near collateral vessels during stroke (52). Moreover, as soon as the occluded vessel was slightly reopened, the overall pressure difference between MCA and ACA territories decreased, thereby reducing the hemodynamic relevance of the LMCs and shifting their flow toward baseline distributions (Fig. 4*C*).

Fig. 4*D* shows the total MCA inflow relative to baseline values (i.e., before stroke) as a function of vessel reopening under intact autoregulation in all arteries. For all networks, the average reperfusion profiles for the 100% and 0% LMC conditions were nearly identical. Interestingly, despite identical model parameters across all networks, the C57BL/6_II_ network exhibited perfusion overshoot in both LMC configurations. This finding suggests that LMC density alone does not drive the perfusion overshoot. Instead, overshoot likely arises from network-specific differences in the impact of myogenic and endothelial regulation. Total ACA inflow remained essentially unchanged under these conditions (*SI Appendix*, Fig. S11*A*).

#### Regulatory determinants of reperfusion overshoot.

To identify the regulatory conditions that may cause perfusion overshoot, we simulated several postischemic dysfunction scenarios (Fig. 4*E*). Namely: i) Vascular elasticity impairment: In a first step, all arteries were assumed to behave as nonregulated vessels, reflecting the global loss of autoregulation seen during stroke. In the second step, the Young’s modulus *E* was reduced to 10% of its baseline value to make vessels more flexible and mimic compromised vascular integrity. The altered Young’s Modulus was applied to the MCA-sided SAs-to-CoW segments and the first two generations of SAs with diameters >40 μm to assess whether localized vessel softening in the affected territory could lead to perfusion overshoot. ii) Myogenic impairment: The myogenic response was modulated by ±50% of its initial value or completely removed. iii) Endothelial impairment: similarly, the endothelial response was modulated by ±50% or fully removed. In both (ii) and (iii), all arteries were assumed to behave actively, and the modifications were only applied to all arteries on the MCA side. For these analyses, we used the four original networks, having demonstrated that the extent of LMCs does not significantly influence the reperfusion profile.

In networks where all arteries, except LMCs, behaved as nonregulated vessels, MCA inflow returned to baseline as the occluded vessel reopened (Fig. 4*F*). This is expected since no active mechanisms were available to induce changes in vessel parameters (see Eq. **4**, $d_{\mathrm{ref}}$ was set constant throughout the simulations). However, when the Young’s modulus of the largest upstream vessels was reduced to 10% of baseline, we observed a clear perfusion overshoot, with relative MCA inflow reaching ~1.3 at 60% reopening. Even though a substantial reduction in Young’s modulus was required to produce a meaningful perfusion overshoot across all networks, this result highlights that elastic vessel properties alone can shape reperfusion dynamics.

Subsequently, we varied the myogenic sensitivity factor $S_{\sigma }$ while keeping the endothelial sensitivity fixed at $S_{\tau }=0.5$, simulating different levels of myogenic responsiveness (Fig. 4*G*). As $S_{\sigma }$ decreased from 6 to 0, the magnitude of MCA inflow during reperfusion increased significantly. For the case of complete myogenic loss ($S_{\sigma }=0$), relative MCA inflow peaked at ~2.1 at 60% reopening, which is in stark contrast to the strongly regulated condition ($S_{\sigma }=6$), where flow returned to baseline. These results identify myogenic impairment as a key contributor to reperfusion overshoot.

If the myogenic response was held constant ($S_{\sigma }=4$), while $S_{\tau }$ was varied from 0.0 (complete loss) to 0.75 (strong endothelial function), we observed the opposite trend (Fig. 4*H*). Increasing $S_{\tau }$ led to a progressive increase in MCA inflow during reperfusion, with relative inflow rising from ~1.0 ($S_{\tau }=0$) to ~1.6 ($S_{\tau }=0.75$) at 60% reopening. This suggests that, contrary to the myogenic response, a strong endothelial response may amplify reperfusion overshoot, likely due to excessive vasodilation in response to elevated shear stress. Thus, endothelial upregulation may contribute to poststroke hyperemia.

Red blood cell (RBC) velocities in MCA capillaries closely mirrored changes in CBF during vessel reopening (*SI Appendix*, Fig. S12). When varying the myogenic sensitivity $S_{\sigma }$, the median RBC velocity remained near baseline levels for high sensitivity (e.g., $S_{\sigma }=6.0$), but increased markedly as myogenic control weakened, reaching nearly twice the baseline value under complete myogenic loss. Similarly, for endothelial sensitivity $S_{\tau }$, RBC velocities increased progressively with stronger endothelial reactivity, though the effect was slightly less pronounced compared to myogenic modulation.

In addition to changes in median RBC velocity, we examined the spatial heterogeneity of RBC velocity during reperfusion by computing the interquartile range (IQR) of RBC velocities across the capillary bed (Fig. 4*I*). Both baseline and strong myogenic regulation ($S_{\sigma }=6.0$) exhibited comparable spatial patterns of RBC velocity variability. Interestingly, in the absence of myogenic response ($S_{\sigma }=0.0$), RBC velocities became more unevenly distributed, indicating higher perfusion heterogeneity. These findings suggest that loss of myogenic reactivity not only elevates capillary flow but also increases RBC velocity heterogeneity. This effect may impair oxygen exchange (69–71) and contribute to tissue dysfunction after stroke.

Together, our findings demonstrate that impaired autoregulation, particularly myogenic dysfunction, is a primary driver of reperfusion overshoot. A reduced Young’s modulus and elevated endothelial sensitivity can further amplify the response. LMCs, on the other hand, are mostly relevant during MCAo and likely by avoiding vascular damage at this stage. These results highlight the intricate interplay between elastic vessel properties and autoregulatory mechanisms in shaping cerebral hemodynamics after stroke and provide plausible explanations for why some patients experience excessive perfusion during reperfusion therapy. Furthermore, higher RBC flow heterogeneity and its association with reduced oxygen extraction efficiency provide another reason why excessive perfusion is linked to poorer outcomes.

## Discussion

Based on our computational framework, we present mechanistic insights into cerebrovascular responses during i) healthy autoregulation, ii) ischemia–reperfusion with altered vascular reactivity, and iii) chronic autoregulatory dysfunction. Importantly, our quantification also addresses perfusion in the capillary bed, which, given its highly interconnected nature, is difficult to study in full detail in vivo.

For healthy autoregulation, the autoregulatory response is not spatially uniform but depends strongly on the artery’s location and baseline intraluminal pressure. The observed shift in peak dilation toward lower-pressure vessel groups with decreasing MAP suggests a hierarchical recruitment of autoregulatory compensation along the arterial tree. Mechanistically, pressure perturbations are not transmitted uniformly throughout the vascular network. Instead, proximal vessels experience larger relative pressure changes during moderate MAP reductions and therefore provide the initial compensation, whereas progressively larger pressure perturbations propagate deeper into the arterial tree as MAP decreases further (*SI Appendix*, Fig. S4). Consequently, autoregulatory demand is redistributed from proximal to distal vascular compartments, with distal vessels becoming increasingly engaged as upstream compensatory capacity becomes exhausted. This interpretation is consistent with previous in silico studies (51) that assumed that vessels of different sizes reach maximal dilation at different MAP levels. Together, these findings suggest that cerebral autoregulation is an emergent network property arising from the spatial propagation of pressure perturbations and the coordinated recruitment of multiple vascular compartments rather than from uniform responses of individual vessels.

Our results align with in vivo studies (4, 5, 17) and underscore the importance of proximal vessels in pressure regulation. Notably, the modeled pressure–diameter responses were also consistent with representative in vivo and ex vivo measurements across vessel sizes (*SI Appendix*, Fig. S6), reproducing the characteristic asymmetric autoregulatory behavior of comparatively modest vasoconstriction during hypertension and stronger vasodilation during hypotension. This provides additional support for the physiological plausibility of the autoregulatory formulation.

Importantly, hierarchical recruitment of autoregulatory responses does not imply that all vascular compartments contribute equally to global flow regulation. Although DAs become increasingly engaged at lower MAP levels and contribute substantially to local vascular resistance, their distributed downstream organization within many parallel penetrating vascular territories limits their influence on total cerebral blood flow. In contrast, upstream SAs and SA-to-CoW segments supply large shared downstream vascular territories, such that relatively small resistance changes propagate through substantial portions of the vascular network and exert stronger control over total cerebral blood flow and the autoregulation curve. These findings identify proximal surface arteries as dominant network control points for cerebral blood flow regulation, aligning with recent theoretical analysis by Ji et al. (72).

More broadly, these results suggest that impairments in different vascular compartments may have pressure-dependent functional consequences, with proximal vessel dysfunction potentially affecting compensation during mild perturbations and distal vessel dysfunction becoming increasingly important during severe hypotension or ischemic conditions. This hierarchical organization of autoregulatory recruitment may provide a useful framework for future experimental studies investigating segment-specific cerebrovascular dysfunction across disease states.

Interestingly, strain-dependent variability in DA density altered this response. Networks with lower DA density required larger and spatially more widespread diameter changes to maintain perfusion. This suggests that a high DA density enhances autoregulatory capacity, which may provide greater resilience against autoregulatory impairments during stroke. While differences in DA density have been reported across mouse strains (53), it remains unknown whether DA density also varies between human individuals and whether such variability contributes to interindividual differences in stroke outcome.

Moreover, autoregulatory failure in the territory most affected during stroke can significantly alter the cerebral autoregulation curve. For instance, even a modest 20% drop in MAP, such as may occur during sleep (73, 74), can lead to a 16% reduction in total CBF when all MCA-sided arteries are impaired, compared to only a 4% reduction with intact autoregulation. This loss of autoregulation propagates downstream, leading to substantial reductions in capillary flow even when only a subset of proximal vessels is affected. These findings suggest that local autoregulatory failure after stroke may increase the brain’s vulnerability to secondary cerebrovascular and neurological complications (75–77) by amplifying the impact of otherwise benign physiological blood pressure variations.

By testing two complementary approaches for autoregulatory impairment, where the number of affected vessels was progressively increased, we implemented a spatially controlled approach to assess the impact of partial loss of regulation. Our findings show that proximal surface arteries play a central role in maintaining stable CBF under both hypotensive and hypertensive conditions. However, even the loss of autoregulation at a few distal arterial branches can cause notable local hypo- or hyperperfusion at the capillary level. Importantly, these local perfusion disturbances can occur despite comparatively modest changes in global cerebral blood flow, highlighting that vessel-specific autoregulatory dysfunction may not be apparent from compartment-level or whole-brain perfusion measures alone.

These results may also have implications for neurovascular coupling (NVC). Since both autoregulation and NVC depend on healthy VSMCs and endothelial cells, ischemic injury that compromises these components may simultaneously impair both mechanisms. In this context, the stroke-induced regional loss of vessel responsiveness may not only impair pressure-driven flow regulation but also reduce the brain’s capacity to match blood supply to neuronal activity (78–81).

Exploiting the benefits of our in silico framework, we showed that LMC extent had limited influence on perfusion in proximal arteries, particularly within the recanalized MCA. This suggests that perfusion in the most upstream regions may be largely independent of the extent of LMCs. Consequently, based on our simulations, the hypothesis that LMCs contribute to maintaining sufficient perfusion to the recanalized MCA and thereby enable it to remain normally regulated may not be fully valid. However, LMC extent clearly contributes to elevating perfusion levels during stroke (52, 82–84) and thereby likely helps protect vessels from autoregulatory impairments (82), which, as shown in the current study, are crucial for healthy cerebral autoregulation. This is further supported by Zhao et al. (47), whose lumped-model simulations predicted that while higher LMC density improved perfusion and oxygen delivery to the ischemic territory, autoregulation still failed locally under stroke conditions.

To identify causes for hyperperfusion during reperfusion, we examined several scenarios involving variations in vessel wall stiffness and autoregulatory function. Both the absence of active regulation, especially when arteries become more flexible (e.g., lower Young’s modulus values), and impaired myogenic response are key drivers for reperfusion overshoot, emphasizing the importance of vasoconstrictive control in preventing excessive perfusion after recanalization. Potential mechanisms to reduce vascular tone and myogenic reactivity during reperfusion include peroxynitrite formation and oxidative stress. Specifically, it was shown that the duration of ischemia and reperfusion (I/R) strongly determines the loss of MCA myogenic activity and tone, with more prolonged I/R producing greater impairment (13, 14, 85). Furthermore, I/R-generated peroxynitrite and protein nitration reduce myogenic tone in MCAs from both the ischemic and contralateral hemispheres, implicating oxidative/nitrative modifications as a bilateral mechanism of myogenic dysfunction and highlighting that vasoreactive impairments are likely not constrained to the ischemic area (86). In consequence, preserving or restoring myogenic responsiveness could be an important therapeutic strategy to limit reperfusion-associated hyperperfusion and downstream injury.

The maximum achievable constriction in our simulations is ~10% due to the definition of the reference diameter, which sets a limit for the smallest attainable diameter. Although previous in vivo and computational studies have reported similar maximum levels of vasoconstriction across a broad MAP range (5, 9, 41, 87, 88), some vascular segments may exhibit stronger responses under extreme hypertensive conditions. In such conditions, our model may underestimate the vasoconstrictive responses at the single-vessel level. However, in most scenarios, this will be compensated by a larger fraction of vessels attaining the largest level of constriction. Only for extreme hypertensive states, our simulations might tend to overestimate perfusion changes, especially at the single-vessel scale.

It should also be noted that in the present framework, nonregulated vessels are represented using a simplified linear elastic pressure–area relationship and therefore do not reproduce the nonlinear stress–strain behavior of passive arterial tissue observed in ex vivo experiments (89–91). Because the primary focus of this study is autoregulatory behavior arising from active compliance modulation rather than detailed passive constitutive mechanics, we considered this approximation appropriate for the present modeling framework. Compliance modulation is defined relative to a baseline reference state, and across the investigated autoregulatory range, the majority of vessels remained within moderate deviations from baseline pressure (*SI Appendix*, Fig. S13), supporting the use of the baseline state as a reasonable local reference condition for the present simulations. Future studies could incorporate more detailed constitutive descriptions of passive arterial mechanics as vessel-specific biomechanical datasets become available. Although such nonlinear formulations may influence the quantitative magnitude of predicted diameter and perfusion changes under large hemodynamic perturbations, we expect the principal network-level autoregulatory trends investigated in the present study to remain preserved.

Additionally, despite the substantial pressure drop along the surface arterial network (Fig. 1*C*), DA and capillary flow velocities are relatively independent of distance from the CoW due to the inverse-modeling calibration (*SI Appendix*, Fig. S14). This spatial homogeneity in microvascular flow should therefore be interpreted as a consequence of the network calibration rather than an independent prediction of the model (*SI Appendix, Microvascular Networks*).

Our study accounts for differences in vascular topology between C57BL/6 and Balb-C mice. However, autoregulatory parameters were assumed to be similar across strains. While strain-specific vascular properties and regulatory sensitivities could be easily incorporated into the model, precise in vivo quantification of such parameters is still lacking, but could be an important extension in future work. This may be particularly relevant given existing evidence of strain-dependent inflammatory responses and vascular remodeling (92, 93). Importantly, such refinements are unlikely to affect our overall conclusions on the role of different arterial segments for healthy autoregulation and the consequences for impaired autoregulation on healthy brain function.

Given that reperfusion injury and failure remain frequent and clinically relevant complications in stroke treatment, there is a strong need to explore new therapeutic strategies. Our simulations suggest that preserving or restoring myogenic tone may present a promising therapeutic strategy to prevent hyperperfusion, reduce the risk of hemorrhagic complications, and improve stroke outcomes following recanalization (85, 86). More broadly, these findings highlight the importance of assessing vascular functional recovery after reperfusion therapy, as persistent vasomotor dysfunction may compromise long-term perfusion stability even after successful vessel reopening.

This in silico study incorporates static autoregulatory mechanisms within realistic cerebrovascular networks to investigate how individual arteries contribute to cerebral blood flow regulation and how the failure of these mechanisms alters perfusion across the microvasculature. A central finding is that cerebral autoregulation cannot be fully understood as a property of isolated vessels. Rather, it emerges from interactions between vascular control mechanisms and vascular network architecture. Our results identify surface arteries as key regulators of global blood flow, demonstrate that arterial density influences overall autoregulatory capacity, and show that even localized autoregulatory impairments can produce substantial downstream alterations in capillary perfusion. Furthermore, the simulations suggest that variability in reperfusion overshoot is likely driven by differences in autoregulatory function rather than solely by the extent of collateral circulation. Together, these findings indicate that cerebral autoregulation is a spatially distributed network phenomenon in which vascular topology and local vessel behavior jointly determine tissue perfusion. By incorporating autoregulation at single-vessel resolution, the framework provides mechanistic insight into how localized vascular dysfunction propagates through the cerebrovascular network and may produce disproportionately large effects on tissue perfusion despite relatively modest changes in global cerebral blood flow.

Importantly, impaired vascular tone is not unique to stroke. A growing body of evidence shows that myogenic dysfunction also occurs in other conditions, such as aging, hypertension, diabetes, and Alzheimer’s disease, where it contributes to cerebrovascular dysregulation and increased vulnerability to flow disturbances (78, 94, 95). Thus, our results may generally contribute to understanding flow abnormalities in diverse pathologies characterized by loss of vascular reactivity. More generally, the present framework provides a platform to investigate how vascular topology and autoregulatory heterogeneity influence tissue perfusion and vulnerability to flow disturbances across a wide range of cerebrovascular disorders.

### Disclosure of Delegation to Generative AI.

The authors declare the use of generative AI in the writing process. According to the GAIDeT taxonomy (2025), the following tasks were delegated to GAI tools (ChatGPT-5.5) under full human supervision: proofreading and editing, quality assessment, and recommendations. Responsibility for the final manuscript lies entirely with the authors.

## Supplementary Material

Appendix 01 (PDF)

## Data Availability

Simulation code data have been deposited in github/microBlooM (v2.0.0) (https://github.com/Franculino/microBlooM). All other data are included in the article and/or *SI Appendix*. Previously published data were used for this work (52).
