# Supplementary material for: Loss of cerebral autoregulation drives abnormal perfusion patterns after stroke
Source: Proc Natl Acad Sci U S A. 2026 Sep 29;123(40):e2534566123. doi: 10.1073/pnas.2534566123 (PMC13643332; doi:10.1073/pnas.2534566123)
Supplement: Supplementary file 1 [file pnas.2534566123.sapp.pdf]

PNAS

Supporting Information for

Loss of Cerebral Autoregulation Drives Abnormal Perfusion Patterns After Stroke

Chryso Lambride[1, 2, 3*], Mohamad El Amki[1,2], Susanne Wegener[1,2], Franca Schmid[3*]
1 Department of Neurology, University Hospital and University of Zurich, Switzerland,
2 Neuroscience Center Zurich, University of Zurich, ETH Zurich, Zurich, Switzerland
3 ARTORG Center for Biomedical Engineering Research, University of Bern, Bern, Switzerland

*Corresponding authors: Chryso Lambride & Franca Schmid
Email: chryso.lambride@usz.ch & franca.schmid@unibe.ch

This PDF file includes:

Supporting text
Figures S1 to S16
Tables S1 to S7
SI References

Supporting Information Text

Microvascular networks

Our simulations have been performed on four previously generated large, semi-realistic microvascular networks (MVNs)[1] derived from two mouse strains with different collateral extent and arterial penetrating tree density. These variabilities make our MVNs well-suited for investigating how autoregulation is achieved across different microvascular architectures.

The MVNs were reconstructed from in vivo surface arterial (SA) networks of C57BL/6 (LMC-rich) and Balb-C (LMC-poor) mice acquired by two-photon imaging (Figure 1A). SA networks were derived from two C57BL/6 and two Balb-C mice, each denoted with subscripts I and II. Each SA network covers a 3.5 × 3.5 mm² region of the cortical surface in the whisker and hindlimb areas, capturing vessels from both the anterior cerebral artery (ACA) and MCA territories (MCA-M4/M5). Descending arteriole (DA) density has been increased to match strain-specific values obtained from in vivo quantifications, with C57BL/6 mice exhibiting higher densities than Balb-C mice[2]. To complete the cortical microvascular architecture, ascending venules (AVs) were added, and penetrating trees were interconnected through a synthetic capillary bed, represented by a stacked honeycomb network. The penetrating trees span cortical depths ranging from approximately 50-750 µm below the cortical surface, while the reconstructed capillary bed extends to depths of ~800µm. Additional arteries (SAs-to-CoW) are included to connect the MCA- and ACA-sided SAs to a common inflow vertex, coming from the circle of Willis (CoW). In agreement with experiments[3], occlusion for stroke simulations is induced at the MCA-M2 bifurcation located along the MCA-sided SAs to CoW arteries. To reduce uncertainty in vessel diameters and ensure agreement with in vivo flow characteristics, an inverse modelling approach was applied to all networks[1]. Specifically, vessel diameters were iteratively adjusted to reproduce experimentally observed red blood cell velocity ranges in selected SA segments as well as across all DA and AV root vessels. This procedure constrains the network haemodynamics using experimentally motivated flow conditions and promotes comparatively homogeneous microvascular perfusion across the simulated domain (Figure S14). A detailed description of MVN generation is provided in[1].

Modification of Leptomeningeal Collateral (LMC) Density

To investigate the isolated role of leptomeningeal collaterals (LMCs) during stroke and reperfusion, additional network configurations were generated by modifying the collateral density of the original networks. For each network, two configurations were considered: one with abundant collaterals (100% LMCs) and one without collaterals (0% LMCs). In the 100% LMC Balb-C configuration, LMCs were added to match the average density observed in C57BL/6 mice by connecting SAs near the watershed border to nearby counterparts in adjacent territories, following Epp *et al.*[1] Apart from the number of LMCs, all network properties remained unchanged between paired configurations

Blood flow model

The microvasculature can be represented as a graph composed of edges and vertices. Each edge represents a blood vessel, and each vertex denotes a junction connecting at least two vessels. As is commonly employed[4–10], blood flow is computed using Poiseuille's law. This approach is appropriate because in the microcirculation, both the Reynolds number (Re < 1) and the Womersley number (Wo < 1) are low, ensuring laminar and quasi-steady flow.

The blood flow rate q_{ij} through each edge e_{ij} was computed as:

$$q_{ij} = \frac{\pi\, d_{ij}^4}{128\, L_{ij}\, \mu_p\, \mu_{rel,ij}}\,(p_i - p_j), \tag{1}$$

where d_{ij} and L_{ij} are diameter and length of the vessel associated with edge e_{ij}. $\mu_p$is plasma viscosity, and $\mu_{rel,ij}$ is the relative apparent viscosity accounting for the Fåhræus–Lindqvist effect. p_i and p_j are the pressure values at the source and target vertex v_i and v_j of edge e_{ij},We accounted for the impact of red blood cells on flow resistance by incorporating the empirical viscosity model developed by Pries et al[11] to compute $\mu_{rel,ij}$ (*in vitro* version). Given our focus on the global flow field rather than capillary specific perfusion, we neglected phase-separation effects

at bifurcations and assumed a uniform hematocrit $H_{t,ij} = 0.3$. The continuity equation g_i for each vertex v_i:

$$g_i = \sum_{v_k \in \mathcal{N}(v_i)} q_{ik} = 0\,, \tag{2}$$

where $\mathcal{N}(v_i)$ is the set of all neighboring vertices connected to vertex v_i, and Eq. (1) result in a system of linear equations. Solving this system with appropriate boundary conditions yields the pressure and flow field across the entire network.

The MVNs contain a single inflow vertex, located at the most upstream surface pial arteries (SA) connected to the circle of Willis (CoW), and multiple outflow vertices corresponding to the roots of ascending veins (AVs). Pressure boundary conditions of 100 mmHg (inflow) and 10 mmHg (outflow) were applied[1]. The inflow pressure at the CoW was assumed to represent the MAP.

Impact of Alternative Velocity Profile Assumptions

We assumed a parabolic velocity profile typical for laminar pipe flow to estimate the wall shear stress in each vessel (Eq.9, in the main manuscript). Although microvascular flow is expected to exhibit blunted velocity profiles, the present model accounts for the associated bulk rheological effects through the diameter-dependent apparent viscosity formulation proposed by Pries *et al.*[11] Furthermore, the endothelial response depends primarily on relative changes in wall shear stress rather than its absolute magnitude, such that systematic differences introduced by alternative velocity profile assumptions cancel in the stiffness formulation (Eq.8, in the main manuscript). As a result, blunted velocity profiles primarily influence the number of vessels close to the endothelial activation threshold, with only modest effects on the resulting autoregulatory behaviour (Table S4).

Sensitivity Analysis of Autoregulatory Model Parameters

Input parameters for computational models describing biological systems often lack direct experimental validation. Thus, given the uncertainty in the key input parameters for the presented autoregulation model, we performed a parameter sensitivity analysis to quantify the relative influence of each input on the model output. Specifically, we examined the sensitivity of our model to four critical parameters: the myogenic sensitivity factor (S_σ), the endothelial (shear-dependent) sensitivity factor (S_τ), the slope of the sigmoidal compliance-stiffness relationship (G), and the bounds on compliance variation – maximum positive and negative changes in compliance ($\Delta C^{\pm}/C_0$).

We conducted this analysis using the Elementary Effects (EE) method, as originally proposed by Morris[12,13], which enables efficient screening of input factors over a wide parameter space. The EE method has been widely used in computational modeling studies[14] due to its ability to evaluate the effect of each parameter independently, while requiring significantly fewer model evaluations compared to variance-based methods. Here, the input value ranges were defined as ±25% of their respective baseline values, and model outputs were evaluated at three representative levels of mean arterial pressure (MAP): 60, 80, and 120 mmHg. As model outputs, we considered (i) the relative cerebral blood flow (CBF), and (ii) the mean relative diameter changes in three arterial subgroups: surface arteries connected to the Circle of Willis (SAs to CoW), surface arteries (SAs), and descending arteries (DAs). This framework enabled us to systematically identify which parameters most significantly influence vascular behavior under varying perfusion pressures. C57BL/6I and Balb-CII networks were used.

The analysis yielded three sensitivity metrics: the mean (μ_{EE}), the standard deviation (σ_{EE}), and the mean of the absolute value of the EEs ($\mu_{|EE|}$). The μ_{EE} metric assesses the overall influence of each factor on the output, and the σ_{EE} metric measures the variability of these effects, with high σ_{EE} values indicating non-linear effects or interactions with other parameters.

Figure S15A illustrates the autoregulation curve for both networks with 95% confidence intervals at each tested MAP value. Figure S15B shows the $\mu_{|EE|}$ metric versus σ_{EE} metric for the relative CBF. At 80 mmHg, S_σ factor emerges as the most influential factor in both networks as indicated by relatively high $\mu_{|EE|}$ and σ_{EE} values. G parameter is the second important factor at this MAP level. However, at the autoregulatory limits (60 and 120 mmHg), $\Delta C^{\pm}/C_0$ factor becomes dominant. This shift likely reflects the transition of vessel compliance into saturation regions of the

compliance–stiffness curve at extreme MAP values, while at 80 mmHg, compliance remains in the more linear regime, thus diminishing the impact of $\Delta C^{\pm}/C_0$.

We plotted the relative CBF as a function of S_σ, $\Delta C^{\pm}/C_0$ and S_τ to explicitly visualize how the perfusion responds to variations in the most influential parameters (Figure S15C). Within the autoregulatory region (80 mmHg), variations in S_σ have a substantial effect, where higher values lead to greater arterial dilation and restoration of CBF toward baseline levels. In contrast, at the autoregulatory limits (60 and 120 mmHg), changes in S_σ have minimal impact without creating any trends. Meanwhile, $\Delta C^{\pm}/C_0$ does not strongly affect CBF at 80 mmHg but becomes increasingly important at the extreme MAP values. Specifically, higher compliance bounds at 60 mmHg allow for greater vasodilation, while lower compliance bounds at 120 mmHg limit vasoconstriction, both of which lead to elevated CBF. Another noteworthy observation is that, despite varying all input parameters within ±25% of their baseline values, the Balb-C_{II} network did not reach baseline CBF at 80 mmHg for any combination of input values, whereas the C57BL/6_I network was able to restore flow closer to baseline under the same conditions. This finding highlights that physiological differences in network topology also significantly contribute to variability in the model output. Table S5 summarizes the maximum and minimum relative CBF values obtained across the tested parameter space for the two networks, as well as the relative differences between these extrema at each MAP level, calculated as, $(CBF_{max} - CBF_{min})/CBF_{min} \times 100$).

Figure S16 shows the $\mu_{|EE|}$ and σ_{EE} matrices for the mean relative diameter changes across three arterial types: surface arteries connected to the Circle of Willis (SAs to CoW), surface arteries (SAs), and descending arterioles (DAs). The trends observed for SAs to CoW vessels closely mirror those seen in the relative CBF analysis, meaning that S_σ is dominant at 80 mmHg, while $\Delta C^{\pm}/C_0$ is more influential at the autoregulation limits. However, in the case of SAs and DAs, no single dominant factor is evident at 80 mmHg. Interestingly, at 60 mmHg, while the trends for SAs and DAs in the C57BL/6I network remain consistent with those observed in the SAs to CoW group, the BALB/c_{II} network exhibits a different pattern, indicating that diameter changes in these arterial groups are primarily driven by S_σ, followed by $\Delta C^{\pm}/C_0$. Additionally, at 120 mmHg, the influence of all parameters appears to diminish across all arterial types. Tables S6 and S7 summarize the relative differences between the maximum and minimum mean relative diameters, *i.e.*, $(mean(d)_{max} - mean(d)_{min})/mean(d)_{min} \times 100$), across the parameter space for three arterial types at different MAP levels. Results are shown separately for the C57BL/6_I and Balb-C_{II} microvascular networks.

The sensitivity analysis not only clarifies how model outputs are affected by variations in input parameter values but also offers meaningful insight into the functioning of the autoregulatory response. Our results indicate that the myogenic sensitivity factor, S_σ, and the bounds on compliance variation, $\Delta C^{\pm}/C_0$, are critical determinants of model behavior across different MAP levels, whereas the endothelial sensitivity factor, S_τ, and the slope of the compliance–stiffness relationship, G, generally have a more negligible influence on the outcome. Furthermore, we observed that differences in network topology can influence autoregulatory behavior and final outcomes to a notable extent.

Supplementary Figures

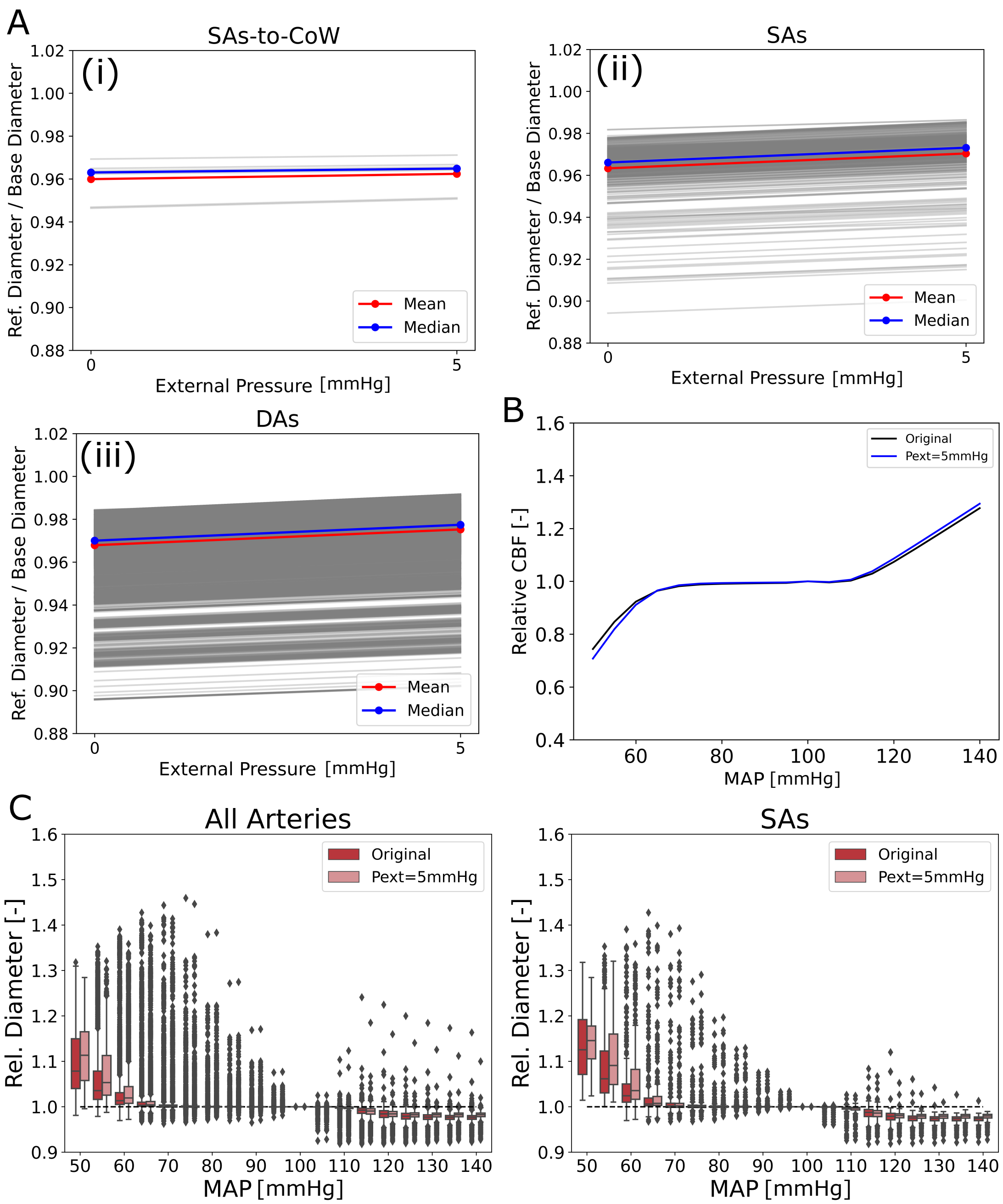

Figure S1. Effect of the extravascular pressure (P_{ext}) on the C57BL/6$_{l}$ network. (A) Ratio of reference diameter to baseline diameter as a function of external pressure (mmHg) across vessel types: (i) surface arteries connected to the Circle of Willis (SAs-to-CoW), (ii) surface arteries (SAs), and (iii) descending arterioles (DAs). (B) Autoregulation curves for the original setup (P_{ext} = *0 mmHg*) and elevated extravascular pressure (P_{ext} = *5 mmHg*). (C) Relative diameter changes as a function of mean arterial pressure (MAP) for the original setup (P_{ext} *=0 mmHg*) and P_{ext} *=5 mmHg*, shown for all arteries (left) and surface arteries (SAs, right).

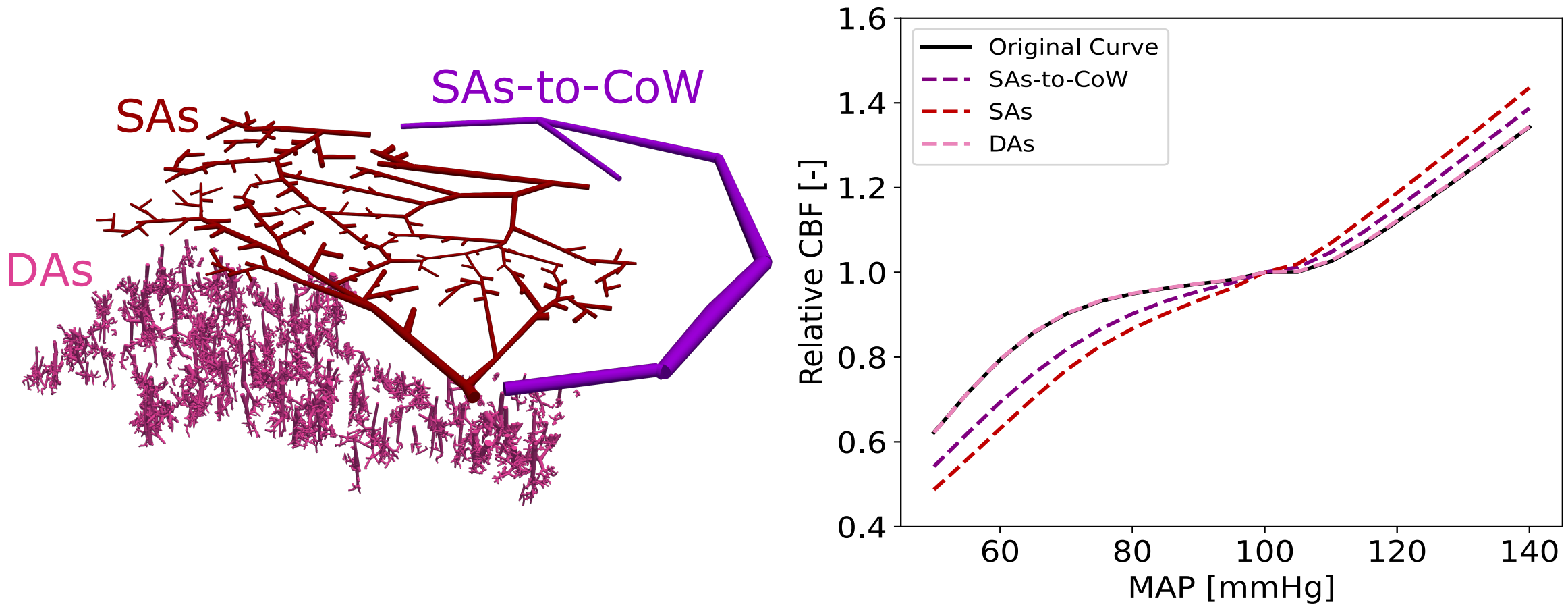

Figure S2. Effect of deactivating autoregulation in different arterial groups on the static autoregulation curve. Relative cerebral blood flow (CBF) as a function of mean arterial pressure (MAP) for four conditions: the fully regulated case (solid black line), and three cases in which autoregulation was independently deactivated in (i) surface arteries connected to the Circle of Willis (SAs-to-CoW, purple dashed), (ii) surface arteries (SAs, red dashed), and (iii) descending arterioles (DAs, pink dashed). Each curve is the mean across the four microvascular networks.

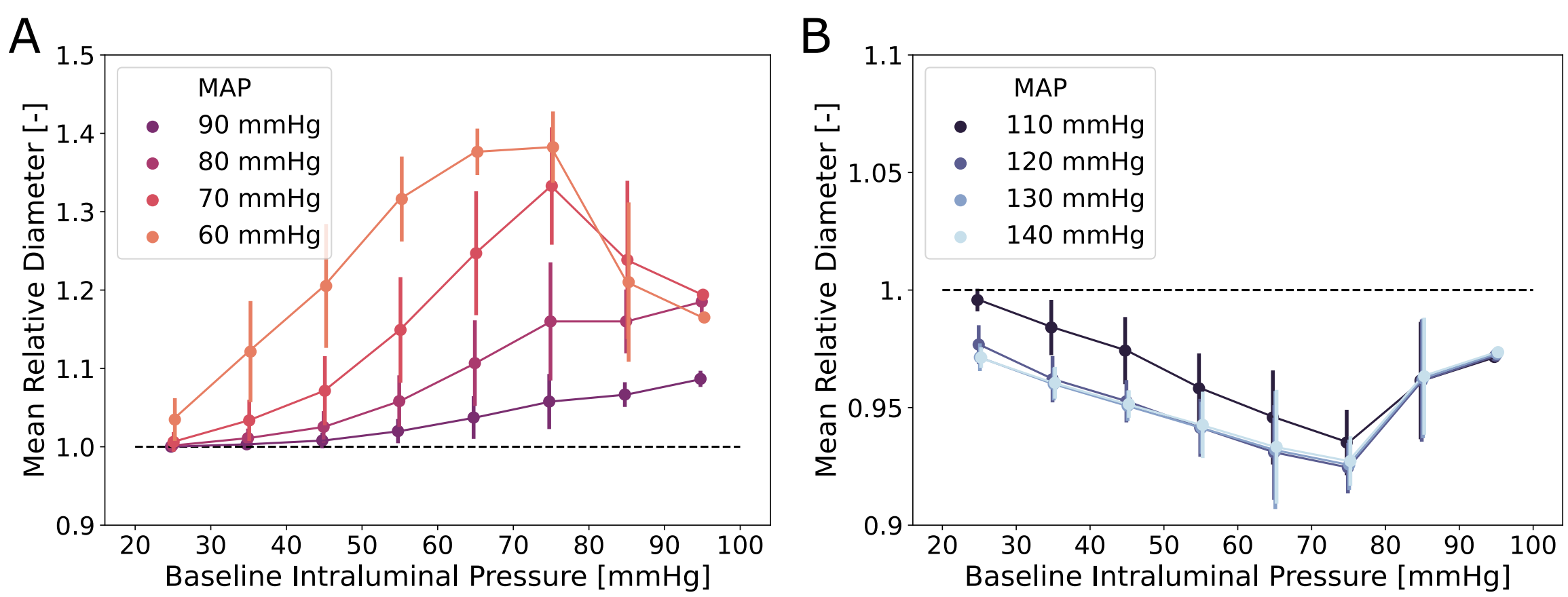

Figure S3. (A) Mean relative vessel diameter as a function of baseline intraluminal pressure under different levels of mean arterial pressure (MAP) reduction (60–90 mmHg). (B) Mean relative vessel diameter as a function of baseline intraluminal pressure under increasing MAP levels (110–140 mmHg). The baseline MAP is 100 mmHg. Results represent the mean ± standard deviation (error bars) computed across all arteries of all four microvascular networks.

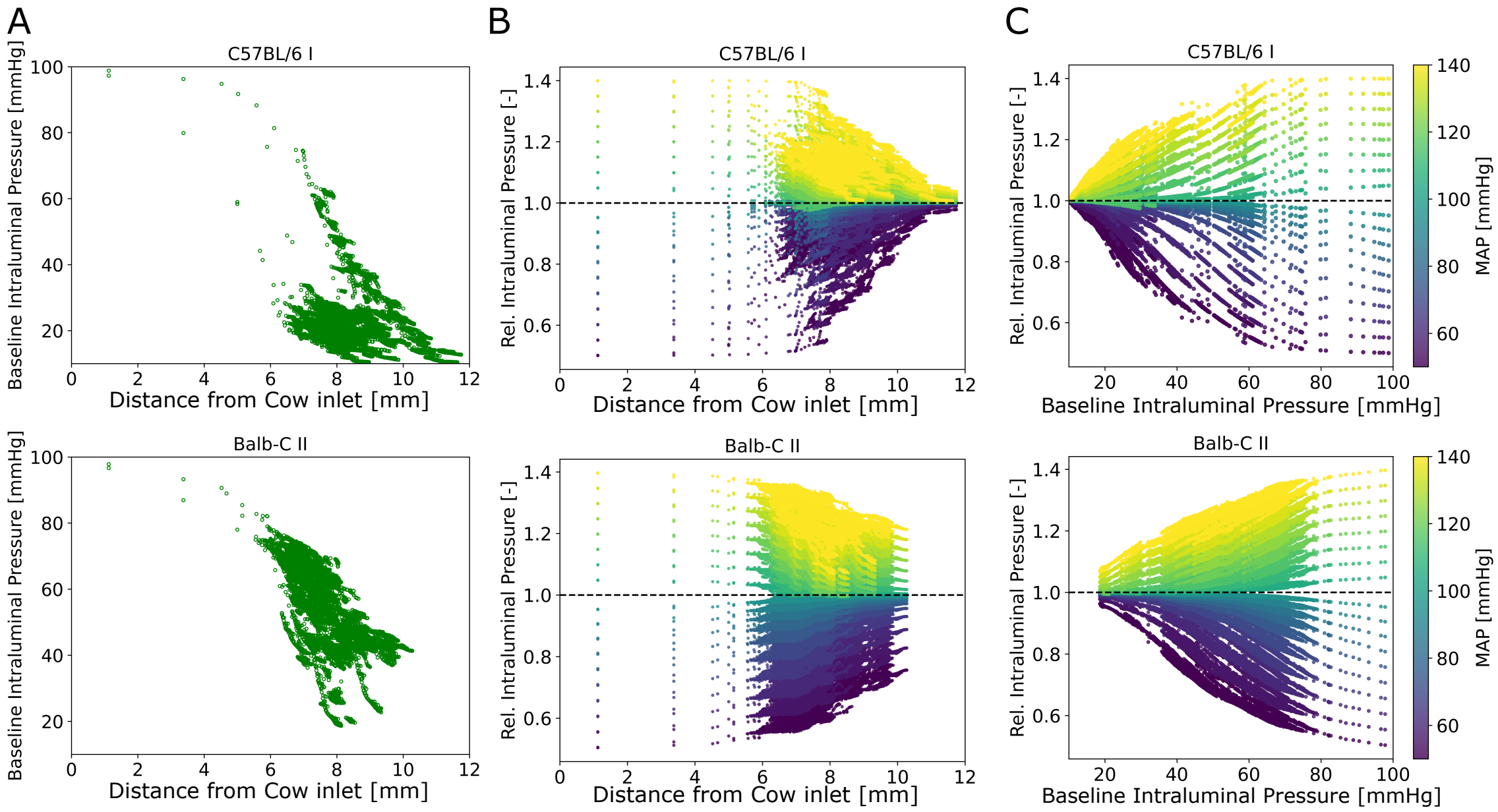

Figure S4: Vessel-specific intraluminal pressure changes support hierarchical recruitment of autoregulation. (A) Baseline intraluminal pressure for all arterial vessels [mmHg] as a function of the distance from the Circle of Willis (CoW) inlet [mm]. (B) Relative intraluminal pressure changes during mean arterial pressure (MAP) variations as a function of the distance from the CoW inlet [mm]. (C) Relative intraluminal pressure changes as a function of baseline intraluminal pressure [mmHg]. The distance represents the cumulative path length from the CoW inlet to the midpoint of each arterial segment. Each point corresponds to one arterial vessel across all simulated MAP conditions. The dashed horizontal line indicates no change relative to baseline conditions. The first row corresponds to the $C57BL/6_{I}$ network and the second row to the $BALB/c_{II}$ network.

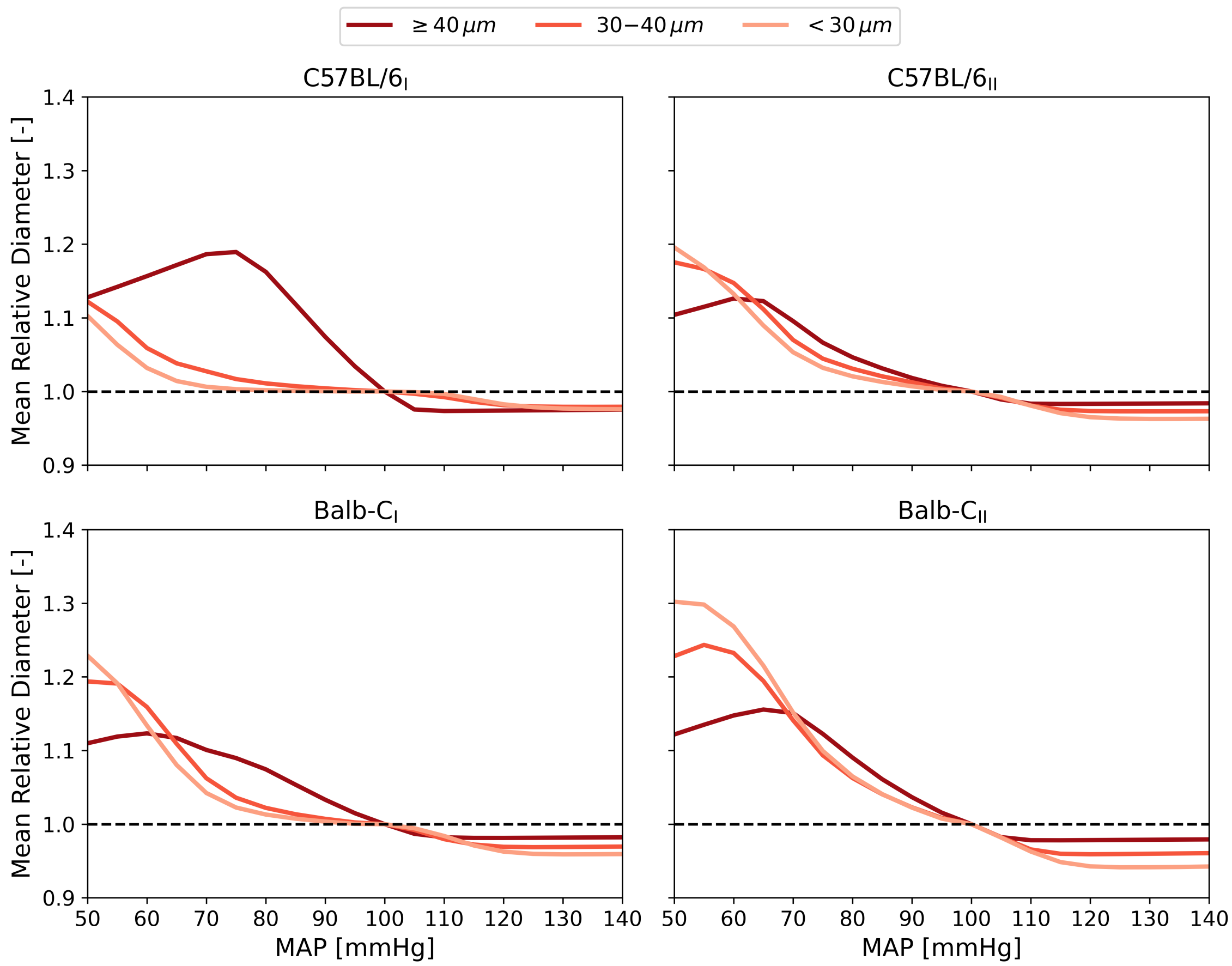

Figure S5: Mean relative vessel diameter as a function of mean arterial pressure (MAP) for three arterial size groups corresponding to the Young's modulus classes used in the autoregulatory model: large arteries (≥40μm), medium arteries (30–40μm), and small arteries (<30μm). Results are shown separately for the four reconstructed vascular networks ($C57BL/6_I$, $C57BL/6_{II}$, $Balb\text{-}C_I$, and $Balb\text{-}C_{II}$). Vessel diameters are normalized to their baseline values at MAP = 100 mmHg (dashed line).

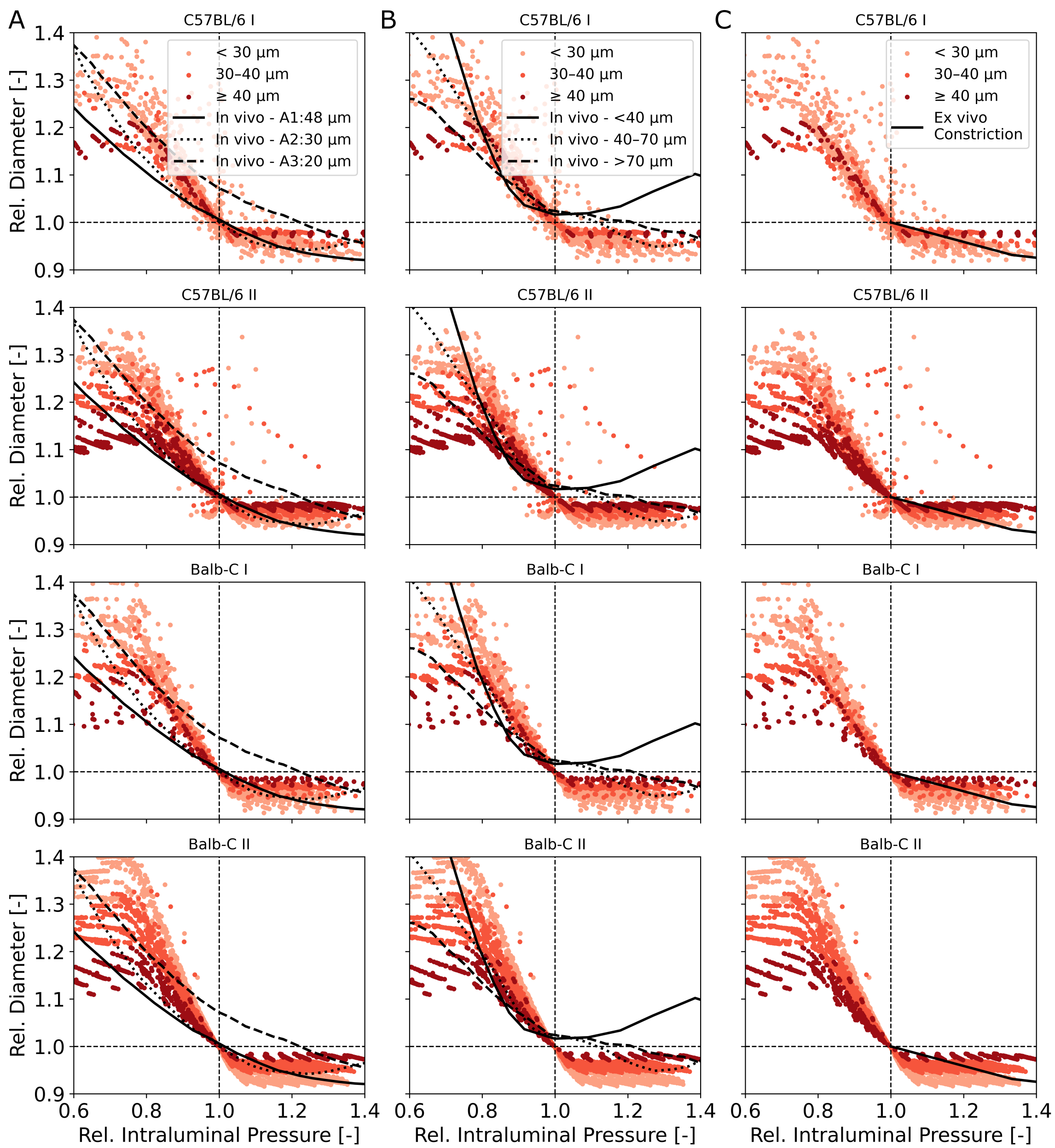

Figure S6: Relative arterial diameter as a function of relative intraluminal pressure across the four reconstructed vascular networks. Rows correspond to the different reconstructed networks (C57BL/6$_{I}$, C57BL/6$_{II}$, Balb-C$_{I}$, and Balb-C$_{II}$), whereas columns show qualitative comparisons with representative experimental pressure–diameter relationships from different studies[15–17]. Arteries are grouped according to the Young's modulus classes used in the model (≥ 40 µm, 30–40 µm, and < 30 µm). Relative intraluminal pressure and relative diameter values are computed for all investigated MAP changes and normalized to baseline conditions. (A) Comparison with *in vivo* pressure–diameter relationships reported by Harper *et al.*[15] Solid, dotted, and dashed black lines represent cerebral arterioles in rats with baseline diameters of approximately 48 µm, 30 µm, and 20 µm, respectively. (B) Comparison with *in vivo* pressure–diameter relationships reported by Klein *et al.*[16] Solid, dotted, and dashed black lines represent cerebral arterioles in piglets with baseline diameters of <40 µm, 40–70 µm, and >70 µm, respectively. (C) Comparison with representative *ex vivo* active pressure–diameter relationships reported by Pires *et al.*[17] in the presence of Ca^{2+}.

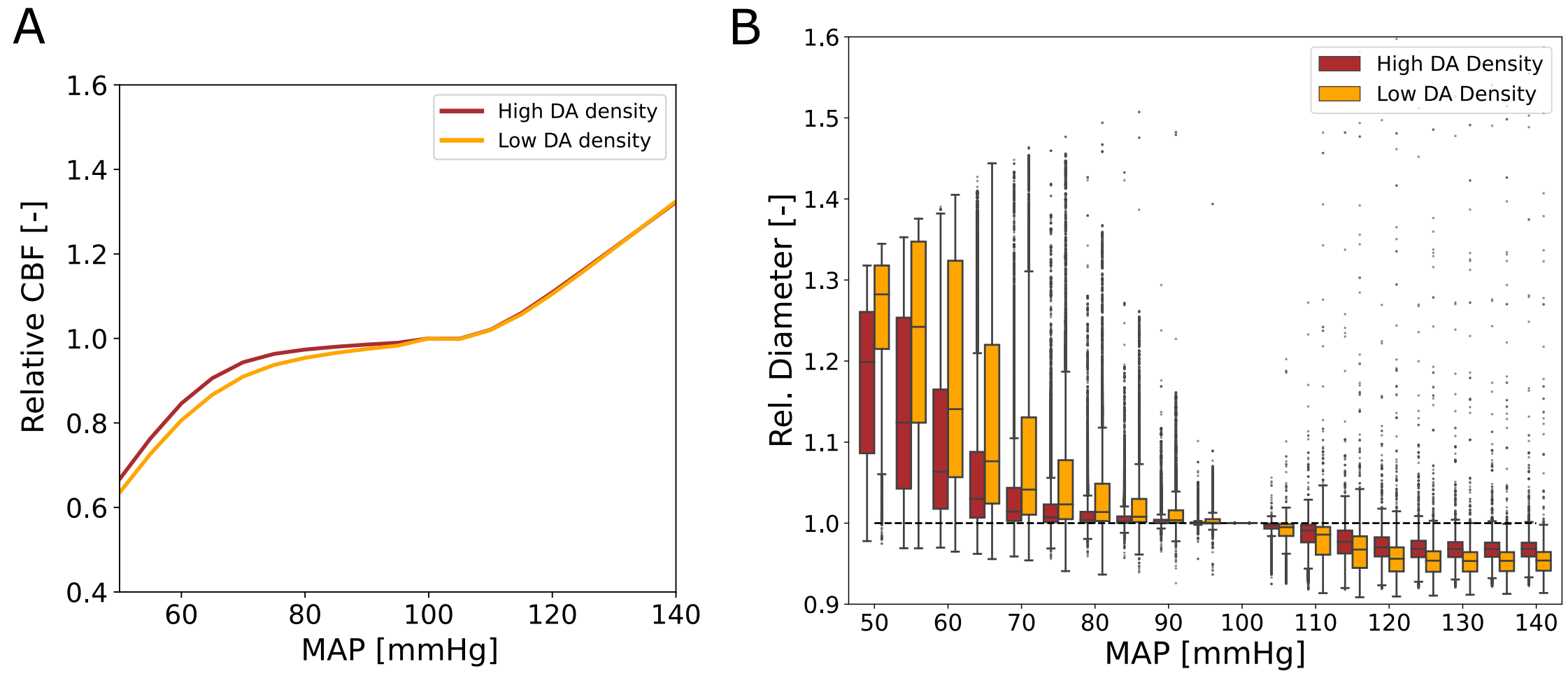

Figure S7. (A) Mean relative cerebral blood flow (CBF) as a function of mean arterial pressure (MAP) for microvascular networks with high and low descending arteriole (DA) densities. (B) Distribution of relative arterial diameters across all vessels in the two configurations, shown in function of MAP. All values are reported relative to their respective baseline values at 100 mmHg MAP. Results are pooled across all four networks for low and high DA density, respectively.

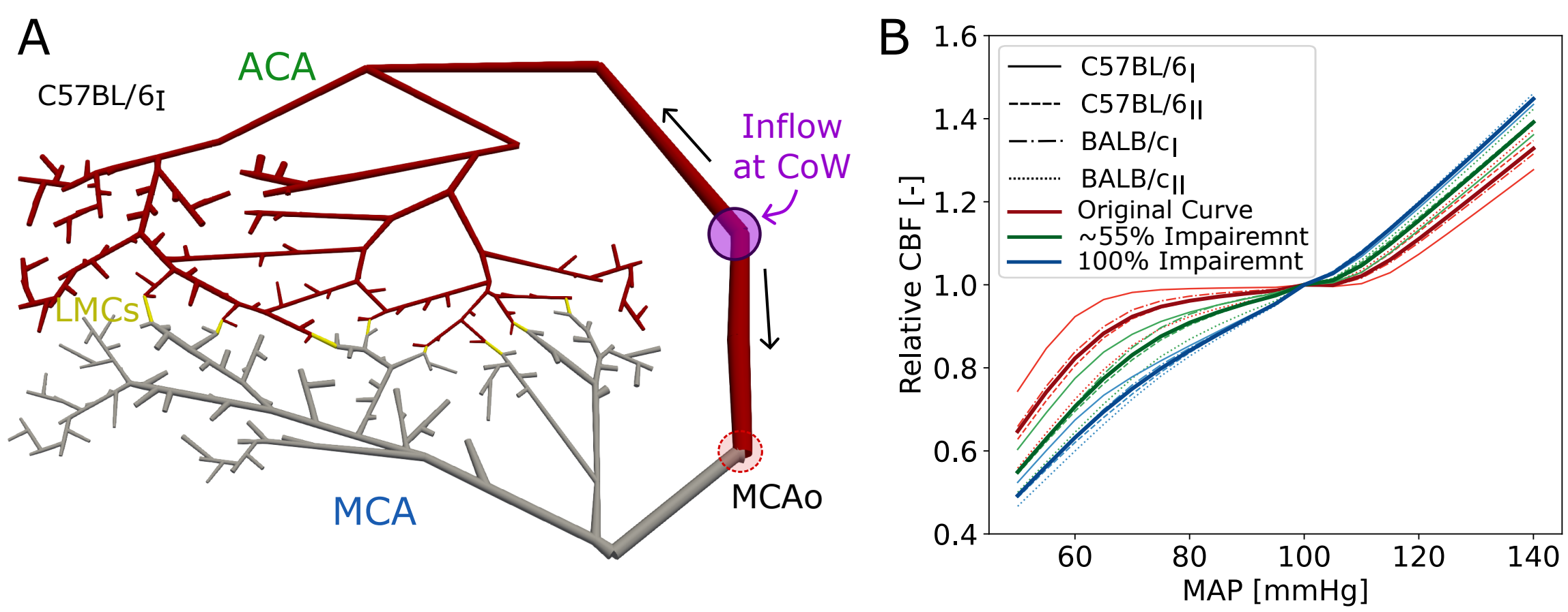

Figure S8. (A) Microvascular network (C57BL/6_I) illustrating the distribution of passive arteries (gray) and actively regulating vessels (red). The site of the previously simulated middle cerebral artery occlusion (MCAo) is indicated by the dashed red circle. For the fully impaired case, autoregulatory impairment extends from the MCAo location throughout the MCA territory, reaching up to the leptomeningeal collaterals (LMCs, shown in yellow). (B) Relative cerebral blood flow (CBF) normalized to baseline values as a function of mean arterial pressure (MAP), comparing fully regulated (original, red curves) and fully impaired (blue curves), and partially impaired (~55% impairment, green curves) conditions across all four networks. The solid thicker lines represent the average curves across all networks for each condition.

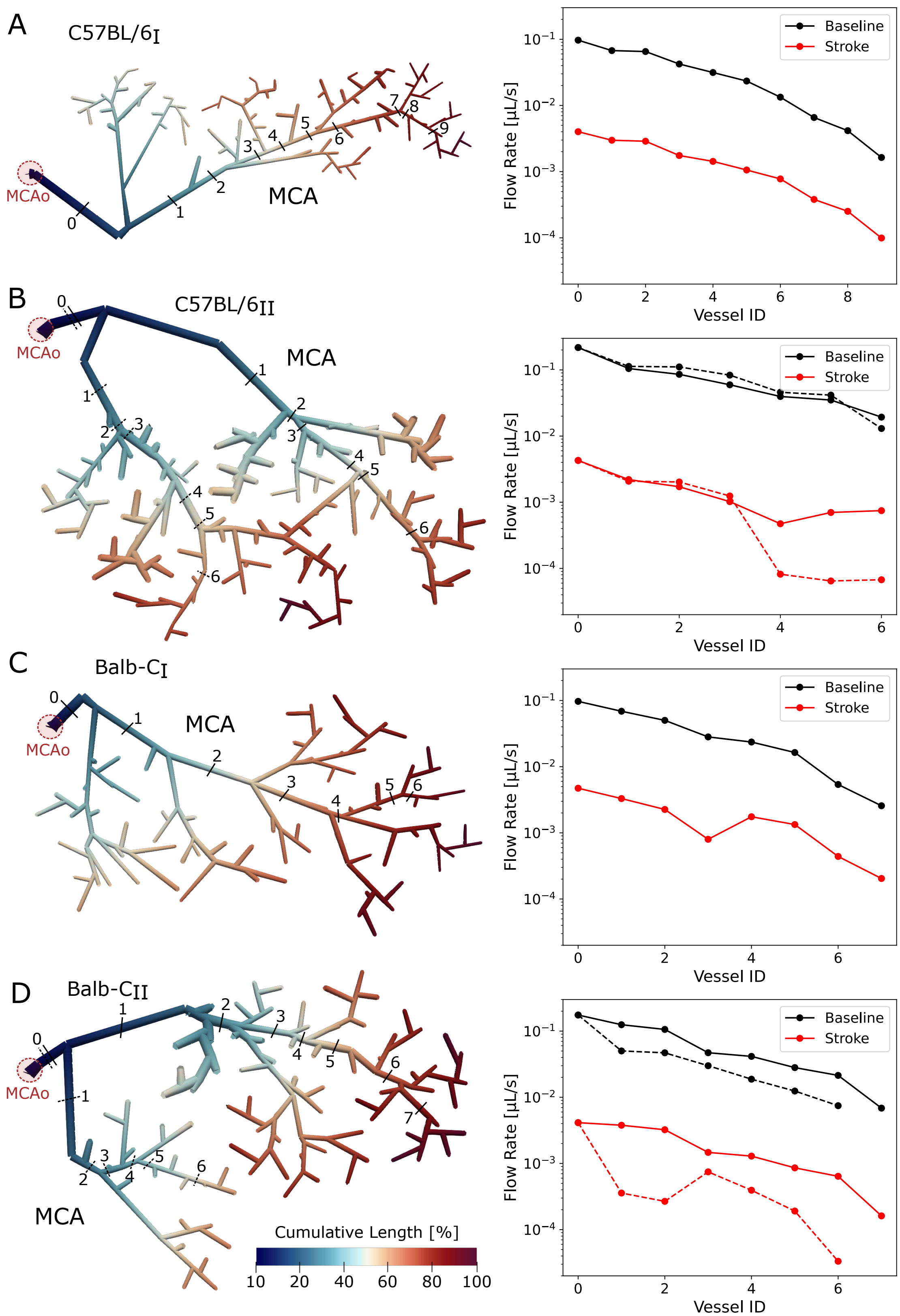

Figure S9. Visualization of cumulative vessel length and corresponding flow rates under baseline and stroke conditions. (A–D) Left: Middle cerebral artery (MCA)-sided vasculature for each microvascular network (C57BL/6$_{I}$, C57BL/6$_{II}$, Balb-C$_{I}$, and Balb-C$_{II}$), colored by cumulative vessel

length, normalized to the maximum value within each network. Selected vessel segments are labeled based on their cumulative distance from the middle cerebral artery occlusion (MCAo, red circle). Right: Exemplary flow rates [μL/s] in each labeled segment under baseline (black) and stroke (red) conditions, illustrating the progressive reduction in perfusion along the vascular path after MCAo.

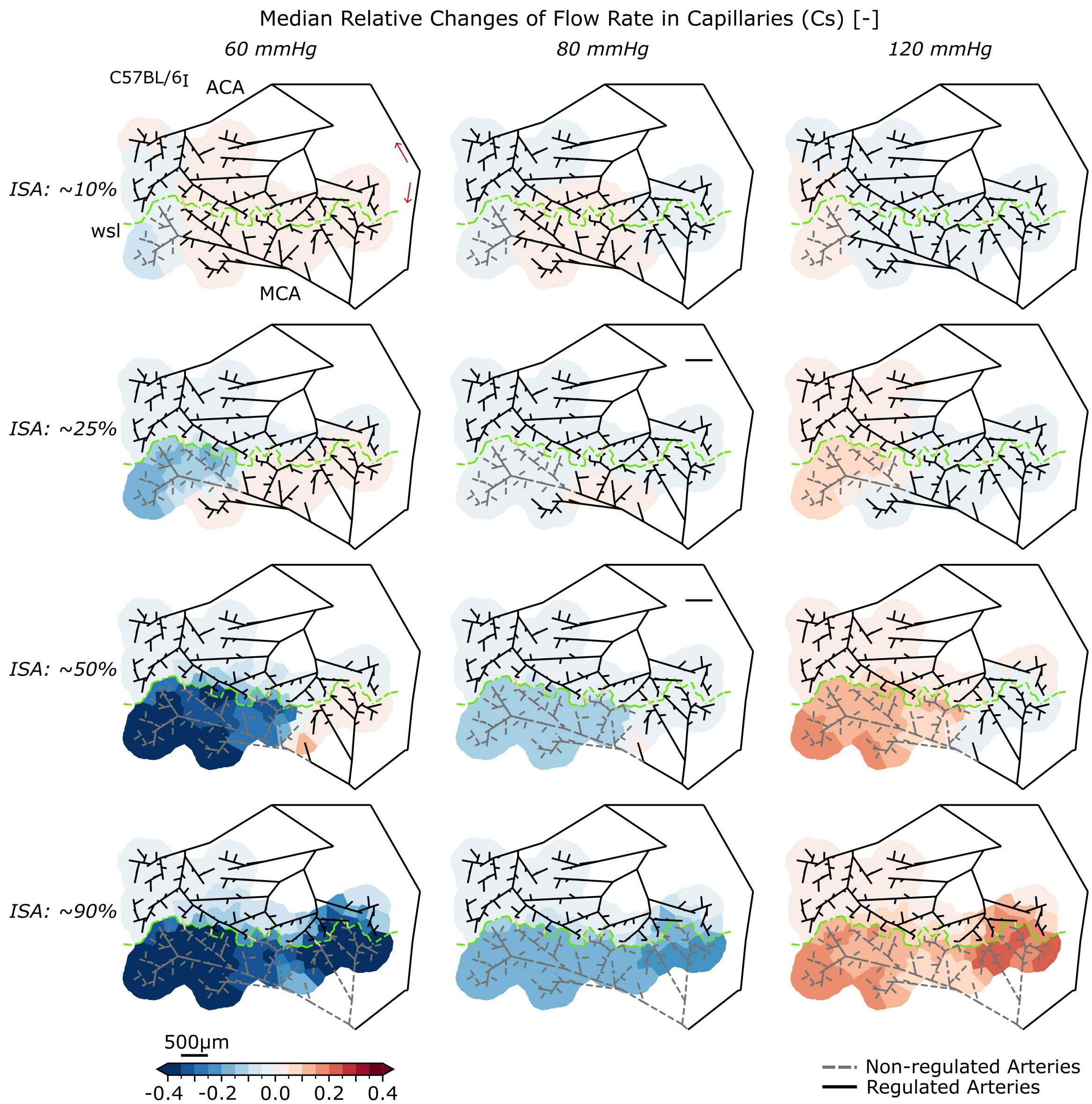

Figure S10. Spatial maps of median relative changes in capillary flow rate at three mean arterial pressure (MAP) levels (60, 80, and 120 mmHg) for four impaired surface area (ISA) scenarios (~10%, ~25%, ~50% and ~90%), as shown in Figure 3D. Passive and regulatory arteries are indicated with dashed and solid lines, respectively. The relative change is computed with respect to the fully regulated case at each pressure level.

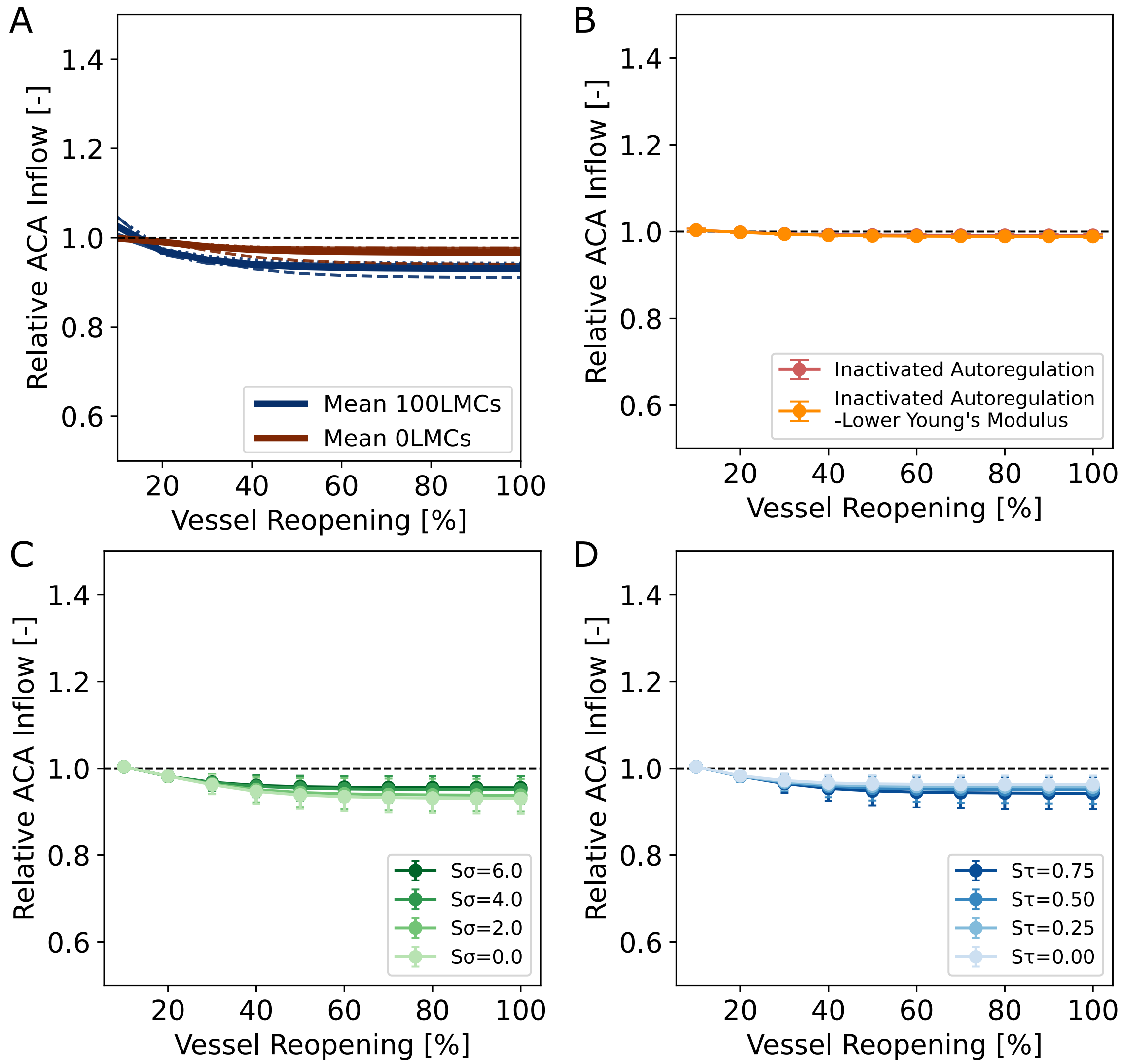

Figure S11. (A) Relative ACA inflow as a function of vessel reopening (from 10% to 100% of baseline diameter), shown for each network with distinct line styles and for both 0% and 100% LMC conditions. Bold lines indicate the average across all networks for each condition. Relative ACA inflow as a function of vessel reopening under altered regulatory mechanisms: (B-D) Relative ACA inflow as a function of vessel reopening under altered regulatory mechanisms: (B) the absence of active autoregulation (passive behavior), with and without reduced Young's modulus; (C) active autoregulation with varying myogenic sensitivity (S_σ = 0, 2, 4, 6); (D) active autoregulation with varying endothelial sensitivity (S_τ= 0, 0.25, 0.5, 0.75). All curves show mean ± SD across networks. Young's modulus and sensitivity factors (S_σ and S_τ) were modified only in arteries within the ischemic territory (*i.e.*, MCA-sided arteries).

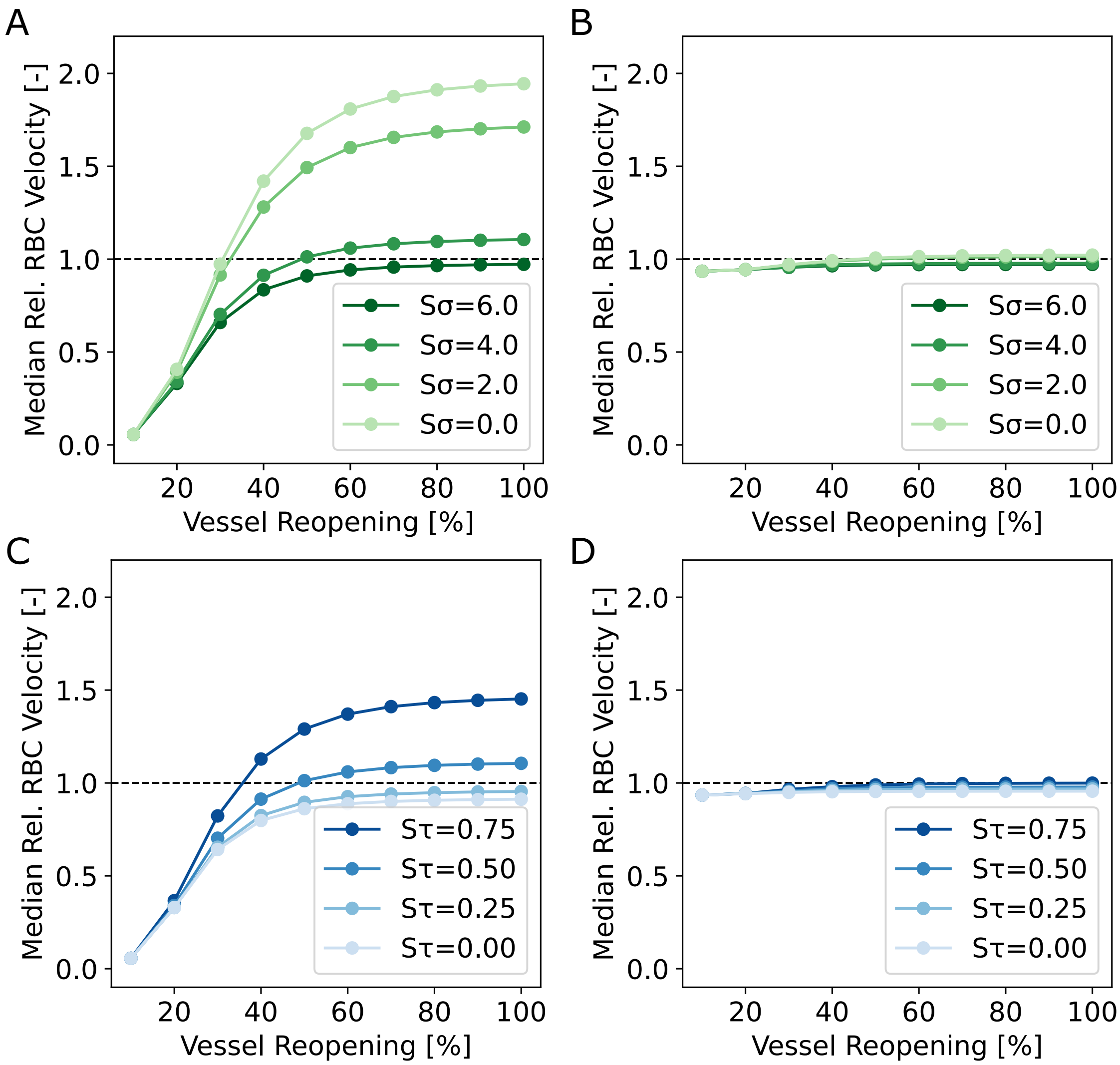

Figure S12. Median RBC velocity relative to baseline as a function of vessel reopening under different regulatory conditions. (A, B) Effects of myogenic sensitivity (S_σ = 0, 2, 4, 6) on median RBC velocity in MCA (A) and ACA (B) capillaries. (C, D) Effects of endothelial sensitivity (S_τ= 0, 0.25, 0.5, 0.75) on median RBC velocity in MCA (C) and ACA (D) capillaries. All curves show the mean across all networks. Sensitivity factors (S_σ and S_τ) were modified in MCA-sided arteries.

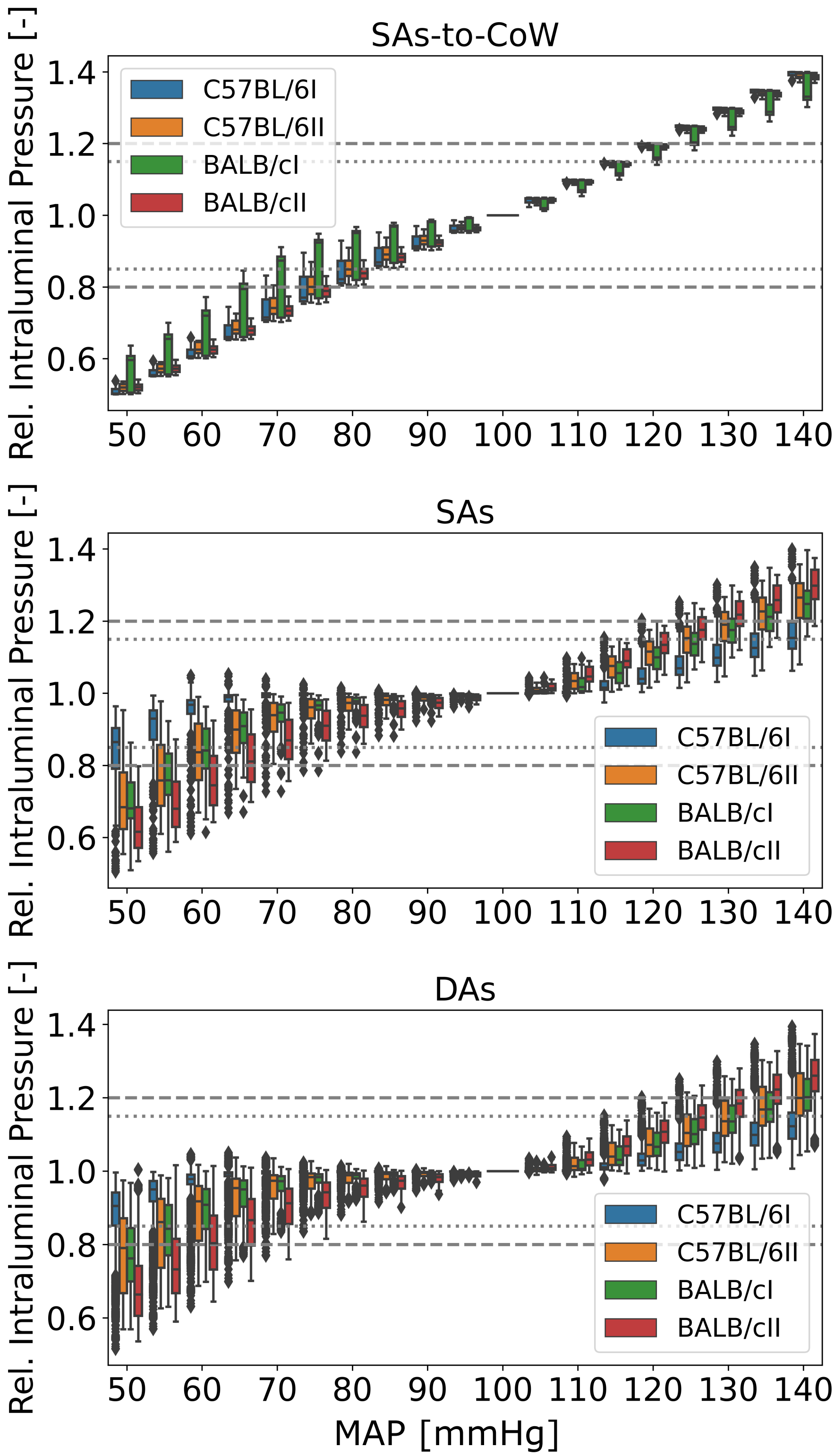

Figure S13: Relative intraluminal pressure normalized to baseline as a function of mean arterial pressure (MAP) across all simulated vascular networks and vessel compartments. Boxplots are shown for surface arteries connected to the Circle of Willis (SAs-to-CoW), surface arteries (SAs), and descending arteries (DAs). Results are presented for the four reconstructed vascular networks (C57BL/6_{I}, C57BL/6_{II}, BALB/c_{I}, and BALB/c_{II}). Dashed horizontal lines indicate ±20% deviations from baseline pressure, while dotted lines indicate ±15% deviations.

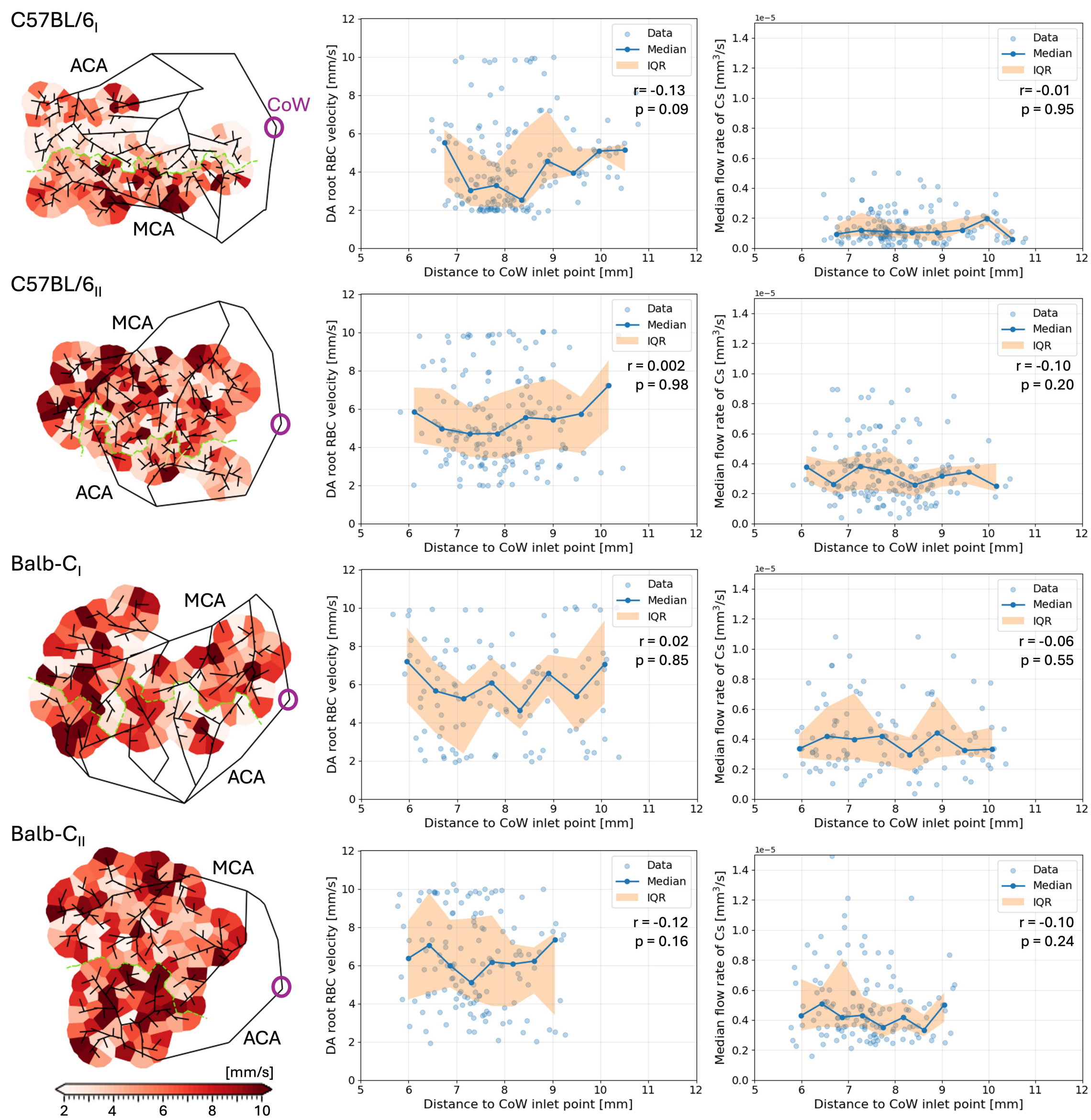

Figure S14: Spatial distribution and downstream variation of hemodynamic metrics across the four vascular networks. Each row corresponds to one network (C57BL/6$_{I}$, C57BL/6$_{II}$, Balb-C$_{I}$, and Balb-C$_{II}$). Left column: Spatial distribution of descending arteriole (DA) root red blood cell (RBC) velocity (mm/s). Colors indicate RBC velocity values at DA root locations. Black lines represent the surface arterial (SA) network, green dashed lines denote the watershed boundary between the anterior cerebral artery (ACA) and middle cerebral artery (MCA) territories, and the purple circle indicates the Circle of Willis (CoW) inlet node. Middle column**:** DA root RBC velocity as a function of the path length distance from the CoW inlet point. Right column: median capillary (Cs) flow rate (mm^3/s) within the corresponding DA territory as a function of the path length distance from the CoW inlet point. For each DA root, the median capillary flow rate was computed across the capillaries contained within its associated spatial territory (left column). The distance represents the cumulative length along the SA path from the CoW inlet to each DA root. Solid lines indicate binned median values, shaded regions represent the interquartile range (IQR). Reported *r* and *p* values denote the Pearson correlation coefficient and associated p-value, respectively.

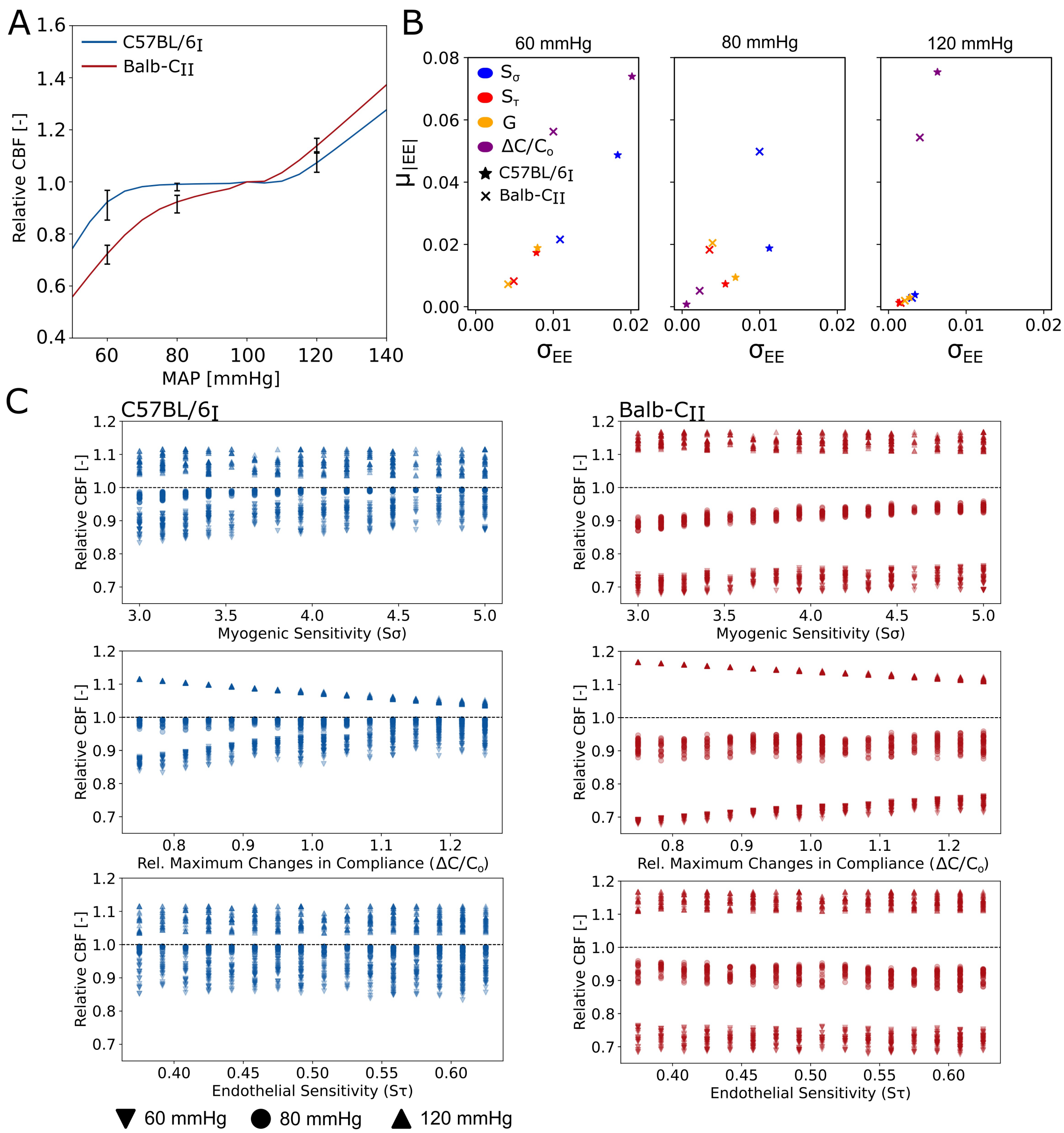

Figure S15. (A) Relative cerebral blood flow (CBF) as a function of mean arterial pressure (MAP), shown with 95% confidence intervals based on variations in input parameters at selected MAP levels (60, 80, and 120 mmHg). (B) Elementary effects (EE) sensitivity plots showing the mean absolute EE ($\mu_{|EE|}$) versus the standard deviation of EE (σ_{EE}) for relative CBF at different MAP values for C57BL/6$_{I}$ and Balb-C$_{II}$ networks. Parameters analysed include the myogenic sensitivity factor (S_σ), the endothelial sensitivity factor (S_τ), the slope of the sigmoidal compliance-stiffness relationship (G), and the bounds on compliance variation ($\Delta C^{\pm}/C_0$). (C) Scatter plots illustrating the dependence of relative CBF on changes in the myogenic sensitivity factor (S_σ), the relative bounds of compliance variations ($\Delta C^{\pm}/C_0$), and the endothelial sensitivity factor (S_τ) for the two networks at the three different MAP levels. Left: C57BL/6$_{I}$. Right: Balb-C$_{II}$. Each data point represents an individual simulation run where parameter values have been varied ±25% around their baseline values.

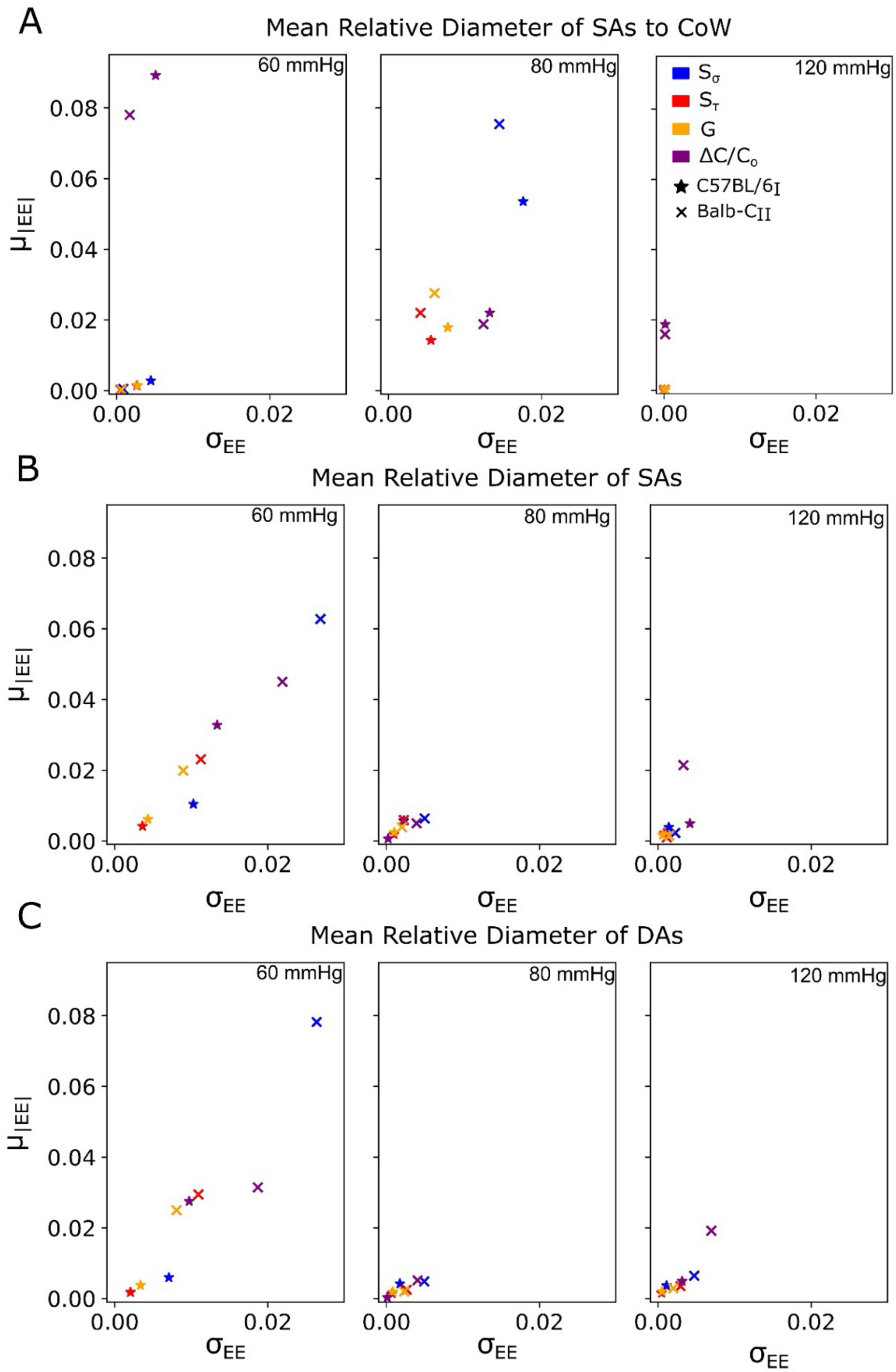

Figure S16. Sensitivity analysis results for mean relative diameters across three arterial types: (A) surface arteries connected to the Circle of Willis (SAs to CoW), (B) surface arteries (SAs), and (C) descending arterioles (DAs). Scatter plots illustrate the mean of the absolute value of elementary effects ($\mu_{|EE|}$) versus their standard deviation (σ_{EE}) at three MAP levels (60, 80 and 120 mmHg) for

the C57BL/6I and Balb-CII networks. Each plot identifies the impact of the four tested parameters (myogenic sensitivity factor, S_σ; the endothelial sensitivity factor, S_τ; the slope of the sigmoidal compliance-stiffness relationship, G; and the bounds on compliance variation, $\Delta C^\pm/C_0$) on diameter responses in the respective arterial group.

Supplementary Tables

Table S1. Parameters used in the autoregulation model and corresponding structural characteristics of the microvasculature. References for parameter values are indicated.

Parameter	Parameter Value	Literature
Autoregulation model parameters		
Myogenic Sensitivity ($S\sigma$)	4.0	[18]
Endothelial Sensitivity ($S\tau$)	0.5	[18]
Sigmoidal Slope (G)	0.1	[18]
Maximum positive compliance changes ($\Delta C^+/C_0$)	10	[18]
Maximum negative compliance changes ($\Delta C^-/C_0$)	0.8	[18]
Characteristics of microvasculature		
Young's Modulus (E) [Pa]		
Arteries (radius ≥ 20 μm)	6.30×10^5	[19]
Arteries (15 μm ≤ radius < 20 μm)	2.60×10^5	[19]
Arteries (radius < 15 μm)	1.60×10^5	[19]
Veins	3.88×10^5	[19]
Capillaries	3.70×10^5	[19]
Vessel wall thickness to diameter ratio (h/D)		
Arteries	0.2	[19]
Veins & Capillaries	0.1	[20]

Table S2. Parameters used to generate new microvascular networks with different descending arteriole (DA) root densities. Δ^{min}_{DA-DA} denotes the minimum distance between two DAs, Δ^{min}_{DA-AV} the minimum distance between DAs and ascending venules (AVs), and Δ^{min}_{AV-AV} the minimum distance between two AVs. These distances were kept constant for both DA root densities (13.4 and 8.9 DAs/mm^2). Table adapted from[1].

$\boldsymbol{\Delta^{min}_{DA-DA}}$	$\boldsymbol{\Delta^{min}_{DA-AV}}$	$\boldsymbol{\Delta^{min}_{AV-AV}}$
200 μm	100 μm	120 μm

Table S3. Relative diameter changes of all leptomeningeal collaterals (LMCs) at stroke conditions, normalized to baseline values (prior to stroke) for each microvascular network (MVN). Mean ± standard deviation (SD) across all networks is also shown.

MVNs	Relative diameter
C57BL/6$_{I}$	
	1.15
	1.15
	1.16
	1.16
	1.15
	1.19
	1.17
	1.16
C57BL/6$_{II}$	
	1.25
	1.23
	1.25
	1.22
Balb-C$_{I}$	
	1.28
	1.27
	1.27
	1.27
	1.27
	1.26
	1.24
Balb-C$_{II}$	
	1.34
	1.26
	1.32
	1.26
Mean ± SD	1.23 ± 0.06
Literature[21]	1.12 ± 0.14

Table S4: Number of surface arteries (SAs) and descending arterioles (DAs) with diameters between 17 and 38 μm exhibiting wall shear stress values below the endothelial activation threshold (0.1 Pa) under baseline conditions for different velocity profile formulations[22,23]. The Koutsiaris *et al*. mean profile corresponds to (k1,k2)=(0.58,22), whereas the additional parameter combinations (0.65,12) and (0.44,34) represent the upper and lower limits derived from the experimentally observed variability reported by Koutsiaris *et al.*[23] Values in parentheses indicate the percentage relative to the total number of vessels in the corresponding vessel class (SA or DA) with diameters between 17 and 38 μm in each network.

**Network**	**Total SAs (17–38 μm)**	**Parabolic profile**	**Tangelder *et al.***	**Koutsiaris *et al*. k_1=0.65, k_2=12**	**Koutsiaris *et al.* k_1=0.58, k_2=22**	**Koutsiaris *et al*. k_1=0.44, k_2=34**
Surface arteries (SAs)						
C57BL/6_{I}	250	6 (2.4%)	5 (2.0%)	5 (2.0%)	1 (0.4%)	0 (0.0%)
C57BL/6_{II}	237	1 (0.4%)	1 (0.4%)	1 (0.4%)	0 (0.0%)	0 (0.0%)
BALB/c_{I}	200	1 (0.5%)	1 (0.5%)	1 (0.5%)	0 (0.0%)	0 (0.0%)
BALB/c_{II}	215	0 (0.0%)	0 (0.0%)	0 (0.0%)	0 (0.0%)	0 (0.0%)
Descending arterioles (DAs)						
C57BL/6_{I}	654	82 (12.5%)	75 (11.4%)	73 (11.1%)	39 (5.9%)	23 (3.5%)
C57BL/6_{II}	714	56 (7.8%)	51 (7.1%)	47 (6.5%)	26 (3.6%)	12 (1.6%)
BALB/c_{I}	558	42 (7.5%)	41 (7.3%)	40 (7.1%)	25 (4.4%)	18 (3.2%)
BALB/c_{II}	591	24 (4.1%)	20 (3.3%)	20 (3.3%)	9 (1.5%)	7 (1.1%)

Table S5. Summary of sensitivity analysis for relative cerebral blood flow (CBF). The table lists the maximum and minimum relative CBF values across the tested parameter space for each mean arterial pressure (MAP) level (60, 80, and 120 mmHg) and for each microvascular network ($C57BL/6_I$ and $Balb\text{-}C_{II}$). The percentage (%) column represents the relative difference between these extreme values.

	Max value	Min value	Relative Change [%]
$C57BL/6_I$			
60 mmHg	0.98	0.83	17.9
80 mmHg	0.99	0.95	4.1
120 mmHg	1.12	1.03	7.9
$Balb\text{-}C_{II}$			
60 mmHg	0.76	0.68	12.9
80 mmHg	0.96	0.87	10.2
120 mmHg	1.16	1.10	5.3

Table S6. Maximum and minimum mean relative diameters and corresponding relative changes (%) for different arterial groups (SAs to CoW, SAs, and DAs) in the $C57BL/6_I$ network at three MAP levels (60, 80, and 120 mmHg). Values reflect the range of model outputs across the tested parameter space in the sensitivity analysis.

	Max value	Min value	Relative Change [%]
60 mmHg			
SAs to CoW	1.23	1.13	8.10
SAs	1.10	1.04	6.18
DAs	1.07	1.01	4.84
80 mmHg			
Sas to CoW	1.20	1.10	9.22
SAs	1.01	1.00	0.85
DAs	1.07	1.00	0.67
120 mmHg			
SAs to CoW	0.98	0.96	1.96
SAs	0.99	0.97	1.08
DAs	0.99	0.98	1.00

Table S7. Maximum and minimum mean relative diameters and corresponding relative changes (%) for different arterial groups (SAs to CoW, SAs, and DAs) in the $Balb\text{-}C_{II}$ network at three MAP levels (60, 80, and 120 mmHg). Values reflect the range of model outputs across the tested parameter space in the sensitivity analysis.

	Max value	Min value	Relative Change [%]
60 mmHg			
SAs to CoW	1.20	1.12	7.02
SAs	1.30	1.16	12.32
DAs	1.33	1.17	13.14
80 mmHg			
SAs to CoW	1.23	1.09	13.03
SAs	1.08	1.06	2.84
DAs	1.07	1.05	2.06
120 mmHg			
SAs-to-CoW	0.98	0.96	1.65
SAs	0.97	0.94	2.67
DAs	0.96	0.93	3.19

SI References

1. Epp, R. *et al.* The role of leptomeningeal collaterals in redistributing blood flow during stroke. *PLoS Comput Biol* **19**, e1011496 (2023).
2. Adams, M. D., Winder, A. T., Blinder, P. & Drew, P. J. The pial vasculature of the mouse develops according to a sensory-independent program. *Sci Rep* **8**, 9860 (2018).
3. Binder, N. F. *et al.* Leptomeningeal collaterals regulate reperfusion in ischemic stroke and rescue the brain from futile recanalization. *Neuron* **112**, 1456-1472.e6 (2024).
4. Boas, D. A., Jones, S. R., Devor, A., Huppert, T. J. & Dale, A. M. A vascular anatomical network model of the spatio-temporal response to brain activation. *NeuroImage* **40**, 1116–1129 (2008).
5. Schmid, F., Reichold, J., Weber, B. & Jenny, P. The impact of capillary dilation on the distribution of red blood cells in artificial networks. *American Journal of Physiology-Heart and Circulatory Physiology* **308**, H733–H742 (2015).
6. Secomb, T. W. Blood Flow in the Microcirculation. *Annu. Rev. Fluid Mech.* **49**, 443–461 (2017).
7. Schmid, F., Tsai, P. S., Kleinfeld, D., Jenny, P. & Weber, B. Depth-dependent flow and pressure characteristics in cortical microvascular networks. *PLoS Comput Biol* **13**, e1005392 (2017).
8. Schmid, F., Barrett, M. J. P., Obrist, D., Weber, B. & Jenny, P. Red blood cells stabilize flow in brain microvascular networks. *PLoS Comput Biol* **15**, e1007231 (2019).
9. Epp, R., Schmid, F., Weber, B. & Jenny, P. Predicting Vessel Diameter Changes to Up-Regulate Biphasic Blood Flow During Activation in Realistic Microvascular Networks. *Front. Physiol.* **11**, 566303 (2020).
10. Schmid, F., Conti, G., Jenny, P. & Weber, B. The severity of microstrokes depends on local vascular topology and baseline perfusion. *eLife* **10**, e60208 (2021).
11. Pries, A. R., Secomb, T. W., Gaehtgens, P. & Gross, J. F. Blood flow in microvascular networks. Experiments and simulation. *Circulation Research* **67**, 826–834 (1990).

12. Campolongo, F., Cariboni, J. & Saltelli, A. An effective screening design for sensitivity analysis of large models. *Environmental Modelling & Software* **22**, 1509–1518 (2007).

13. *Global Sensitivity Analysis: The Primer*. (John Wiley, Chichester, England Hoboken, NJ, 2008). doi:10.1002/9780470725184.

14. Daher, A. & Payne, S. A dynamic multiscale model of cerebral blood flow and autoregulation in the microvasculature. *Applied Mathematical Modelling* **123**, 213–240 (2023).

15. Harper, S. L., Bohlen, H. G. & Rubin, M. J. Arterial and microvascular contributions to cerebral cortical autoregulation in rats. *American Journal of Physiology-Heart and Circulatory Physiology* **246**, H17–H24 (1984).

16. Klein, S. P., De Sloovere, V., Meyfroidt, G. & Depreitere, B. Differential Hemodynamic Response of Pial Arterioles Contributes to a Quadriphasic Cerebral Autoregulation Physiology. *JAHA* **11**, e022943 (2022).

17. Pires, P. W., Dabertrand, F. & Earley, S. Isolation and Cannulation of Cerebral Parenchymal Arterioles. *JoVE* 53835 (2016) doi:10.3791/53835.

18. Daher, A. & Payne, S. A network-based model of dynamic cerebral autoregulation. *Microvascular Research* **147**, 104503 (2023).

19. Salotto, A. G., Muscarella, L. F., Melbin, J., Li, J. K.-J. & Noordergraaf, A. Pressure pulse transmission into vascular beds. *Microvascular Research* **32**, 152–163 (1986).

20. Enouri, S., Monteith, G. & Johnson, R. Characteristics of myogenic reactivity in isolated rat mesenteric veins. *American Journal of Physiology-Regulatory, Integrative and Comparative Physiology* **300**, R470–R478 (2011).

21. Glück, C. *et al.* Pia-FLOW: Deciphering hemodynamic maps of the pial vascular connectome and its response to arterial occlusion. *Proc. Natl. Acad. Sci. U.S.A.* **121**, e2402624121 (2024).

22. Tangelder, G. J. *et al.* Velocity profiles of blood platelets and red blood cells flowing in arterioles of the rabbit mesentery. *Circulation Research* **59**, 505–514 (1986).

23. Koutsiaris, A. G. A velocity profile equation for blood flow in small arterioles and venules of small mammals in vivo and an evaluation based on literature data. *Clinical Hemorheology and Microcirculation* **43**, 321–334 (2009).
